# Nasal administration of mitochondria reverses chemotherapy-induced cognitive deficits

**DOI:** 10.7150/thno.53474

**Published:** 2021-01-01

**Authors:** Jenolyn F. Alexander, Alexandre V. Seua, Luis D. Arroyo, Pradipta R. Ray, Andi Wangzhou, Laura Heiβ-Lückemann, Manfred Schedlowski, Theodore J. Price, Annemieke Kavelaars, Cobi J. Heijnen

**Affiliations:** 1 Laboratories of Neuroimmunology, Department of Symptom Research, Division of Internal Medicine, The University of Texas, M.D. Anderson Cancer Center, Houston, Texas, United States.; 2 School of Behavioral and Brain Sciences and Center for Advanced Pain Studies, The University of Texas at Dallas, Richardson, Texas, United States.; 3 Institute of Medical Psychology and Behavioral Immunobiology, University Hospital Essen, University of Duisburg-Essen, Essen, Germany.

**Keywords:** chemobrain, mitochondria, nasal delivery, nrf2, mesenchymal stem cell

## Abstract

Up to seventy-five percent of patients treated for cancer suffer from cognitive deficits which can persist for months to decades, severely impairing quality of life. Although the number of cancer survivors is increasing tremendously, no efficacious interventions exist. Cisplatin, most commonly employed for solid tumors, leads to cognitive impairment including deficits in memory and executive functioning. We recently proposed deficient neuronal mitochondrial function as its underlying mechanism. We hypothesized nasal administration of mitochondria isolated from human mesenchymal stem cells to mice, can reverse cisplatin-induced cognitive deficits.

**Methods:** Puzzle box, novel object place recognition and Y-maze tests were used to assess the cognitive function of mice. Immunofluorescence and high-resolution confocal microscopy were employed to trace the nasally delivered mitochondria and evaluate their effect on synaptic loss. Black Gold II immunostaining was used to determine myelin integrity. Transmission electron microscopy helped determine mitochondrial and membrane integrity of brain synaptosomes. RNA-sequencing was performed to analyse the hippocampal transcriptome.

**Results:** Two nasal administrations of mitochondria isolated from human mesenchymal stem cells to mice, restored executive functioning, working and spatial memory. Confocal imaging revealed nasally delivered mitochondria rapidly arrived in the meninges where they were readily internalized by macrophages. The administered mitochondria also accessed the rostral migratory stream and various other brain regions including the hippocampus where they colocalized with GFAP^+^ cells. The restoration of cognitive function was associated with structural repair of myelin in the cingulate cortex and synaptic loss in the hippocampus. Nasal mitochondrial donation also reversed the underlying synaptosomal mitochondrial defects. Moreover, transcriptome analysis by RNA-sequencing showed reversal of cisplatin-induced changes in the expression of about seven hundred genes in the hippocampus. Pathway analysis identified Nrf2-mediated response as the top canonical pathway.

**Conclusion:** Our results provide key evidence on the therapeutic potential of isolated mitochondria - restoring both brain structure and function, their capability to enter brain meninges and parenchyma upon nasal delivery and undergo rapid cellular internalization and alter the hippocampal transcriptome. Our data identify nasal administration of mitochondria as an effective strategy for reversing chemotherapy-induced cognitive deficits and restoring brain health, providing promise for the growing population of both adult and pediatric cancer survivors.

## Introduction

Nearly 3 out of 4 cancer patients undergoing chemotherapy suffer from loss of memory, attention, concentration, processing speed, executive and psychomotor function as well as visuospatial skills 1,2. This range of cognitive deficiencies referred to as chemotherapy-induced cognitive impairment, chemobrain or chemofog severely hampers quality of life of patients undergoing treatment as well as of survivors since it significantly impacts their daily activities, social interactions and ability to return to school or work, involving severe financial hardship 3. Advanced neuroimaging analyses have identified structural white and gray matter damage following chemotherapy in patients treated for various types of cancer, including breast 4, colon 5, testicular 6 and lung cancer 7. Cognitive deficits associated with platinum-based therapeutics such as cisplatin have been observed for 5-10 years post-diagnosis in ovarian cancer survivors 8, at least 2 years post-chemotherapy in head and neck cancer survivors 9 and 10-26 years post-treatment in testicular cancer survivors 10,11. However, no United States Food and Drug Administration-approved therapeutic interventions are available to date. We are therefore faced with a critical need to develop an effective strategy for preventing or reversing the cognitive deficits resulting from chemotherapy.

Cisplatin, a platinum-based antineoplastic drug that targets the purine bases of DNA and inhibits replication, transcription, and repair 12, is widely used to treat solid tumors such as head and neck, small and non-small cell lung, breast, testicular and ovarian cancers 13,14. It inhibits mitochondrial electron chain transport complexes I-IV leading to 70% reduced ATP production 15. Cisplatin enters the brain at concentrations that are sufficient to damage neurons in *in vitro* systems and thus can impact healthy neurons and glia negatively influencing brain function, including cognition 16. We have previously established in a mouse model that cisplatin-induced cognitive impairment is associated with compromised cortical white matter integrity, reduced neuronal spine density 17 and synaptic integrity 18,19 and defective synaptosomal mitochondria 17,20. In a more recent study, we demonstrated that nasal administration of mesenchymal stem cells (MSC) restores cisplatin-induced cognitive impairment in mice and our data indicated that these MSC act by repairing neuronal mitochondrial damage. It appeared that autologous MSC work as well as human MSC in our mouse model 21. We also showed previously that treatment of mice with cisplatin leads to a rapid accumulation of mitochondrial p53. Moreover, co-administration of the mitochondrial protectant pifithrin-µ, which selectively inhibits mitochondrial p53 translocation, prevents cisplatin-induced cognitive deficits as well as the associated structural changes in mitochondria and damage to white matter 20. Together these findings indicate that cisplatin-induced accumulation of mitochondrial p53 is the cause of the damage to brain mitochondria that leads to cognitive deficits in response to treatment with this chemotherapeutic.

Mitochondrial dysfunction, characterized by abnormal morphology, impaired bioenergetics, altered mitochondrial dynamics and mitochondrial DNA mutations, has emerged as an underlying mechanism of several pathologies, including neurodegenerative diseases 22,23, cerebral and cardiac ischemia 24,25, traumatic brain injury 26, spinal cord injury 27, cancer, and chemotherapy-induced cognitive impairment 21 and peripheral neuropathy 28. Following cerebral ischemia, astrocytes donate their healthy mitochondria to ischemic neurons with dysfunctional mitochondria to maintain adequate mitochondrial function and survival 29. We recently showed that astrocytes donate their healthy mitochondria and rescue primary cortical neurons damaged by cisplatin *in vitro* 30.

Based on our above-mentioned findings that mitochondrial deficits underlie cisplatin-induced neuronal damage and cognitive deficits, and that healthy mitochondria can be taken up by damaged neurons, we hypothesized that isolated mitochondria from healthy MSC can be used to resolve cisplatin-induced cognitive deficits and the associated structural damage. This would provide an advantage from the perspective of safety because we do not need donation of allogeneic intact cells. We evaluated the effects of nasal administration of human MSC-derived mitochondria on cognition in cisplatin-treated mice by examining executive function, working and spatial memory. We then investigated distribution of mitochondria in meninges and brain upon nasal administration and their impact on integrity of white matter, synapses and synaptosomal mitochondria. Finally, we explored transcriptomic changes in the hippocampus to identify key canonical pathways, transcriptional regulators and genes that contribute to the cognitive restoration triggered by two nasal administrations of mitochondria.

## Results

### Nasally delivered human MSC-derived mitochondria resolve cisplatin-induced cognitive impairment

**Figure 1:**
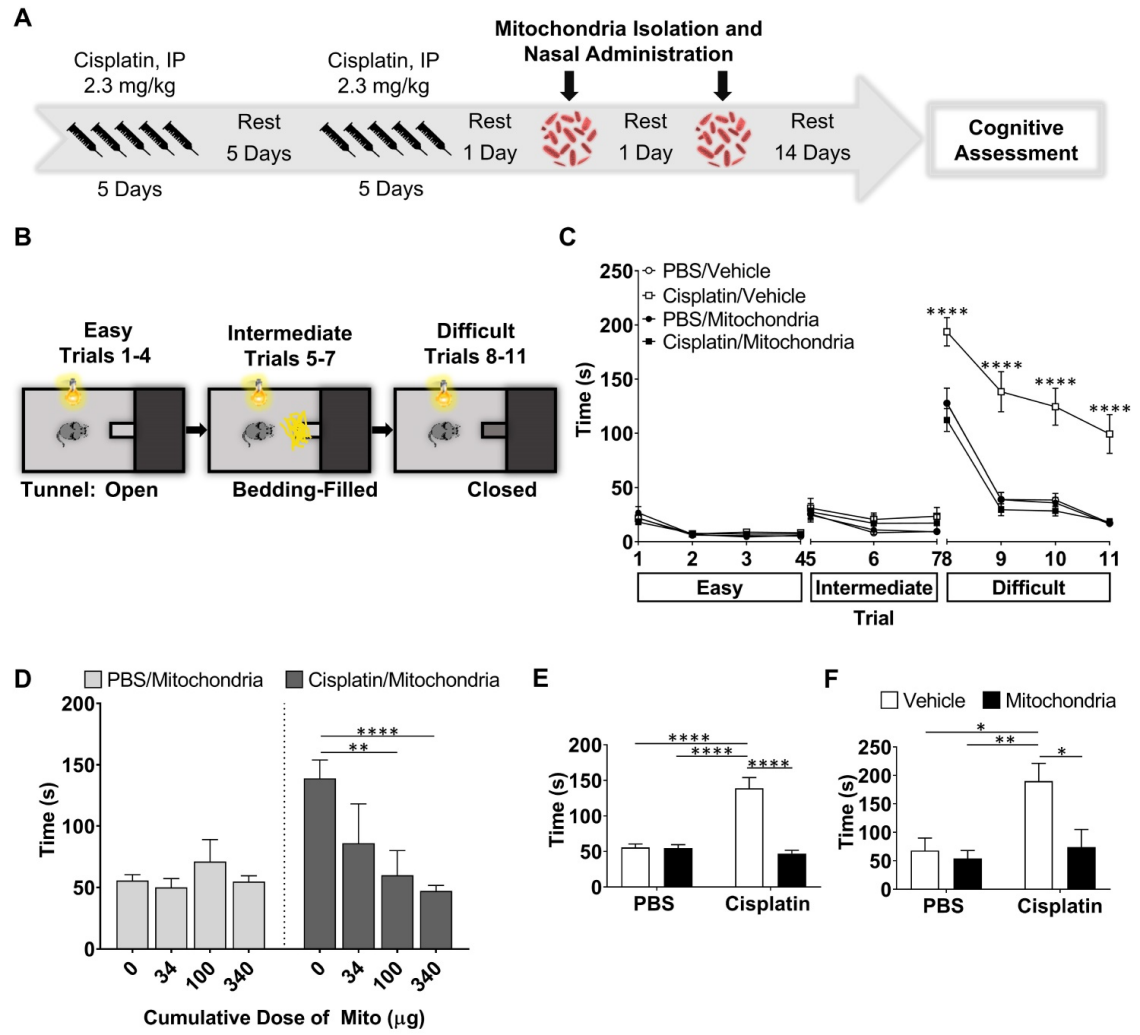
** Nasal administration of mitochondria derived from human MSC resolves cisplatin-impaired executive function.** (A) Schematic representation of cisplatin-treatment regimen, mitochondria-based therapeutic strategy and cognitive testing. Mice were intraperitoneally injected with cisplatin at 2.3 mg/kg for 5 consecutive days, followed by 5 days of rest and another 5 days of cisplatin injection. On the second and fourth day following the last cisplatin injection, mitochondria were freshly isolated from human MSC and administered intranasally. 14 days later, their cognitive behavior was assessed. (B) Schematic representation of PBT for evaluating executive function. Mice in the bright chamber were subject to access the dark chamber at three levels of complexity - an open tunnel (easy trials 1-4), bedding-covered tunnel (intermediate trials 5-7) and plugged tunnel (difficult trials 8-11). (C) The time taken to enter the dark chamber was measured. Cisplatin-treated mice were either slow or failed to unplug the tunnel entry in the difficult trials. Mice, nasally administered with mitochondria performed efficiently (n = 16-22). (D) The restoration of the executive function was determined at 34, 100 and 340 µg of mitochondrial protein with significant effects at 340 µg, in the difficult trials (n = 4-22). Cisplatin-impaired executive function was resolved by nasal administration of mitochondria in both (E) male (n = 16-22) and (F) female mice (n = 6) as seen in the mean time taken to remove the plug and enter the dark chamber. Results are expressed as mean ± SEM; Two-way ANOVA with Tukey's post hoc analysis *p ≤ 0.05; **p ≤ 0.01; **** p ≤ 0.0001.

**Figure 2:**
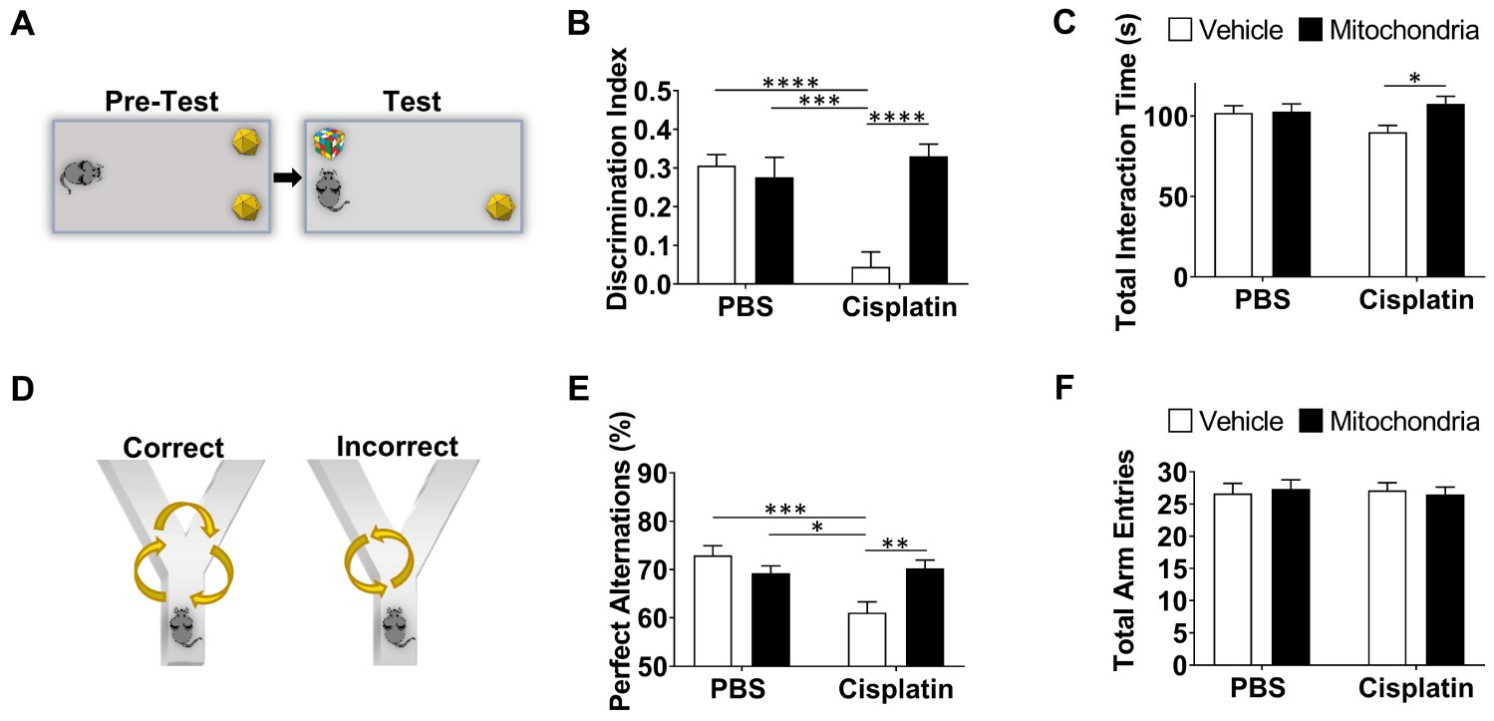
** Nasal administration of mitochondria resolves cisplatin-impaired working and spatial memory.** (A) Schematic representation of NOPRT for evaluating spatial and working memory of mice. Mice were introduced to 2 identical objects during the training period, subsequently returned to their cages and then replaced in the testing arena with the familiar object at the original location and a new object at the opposite corner. (B) The mice movement and time spent with each object were tracked. The discrimination index was calculated as (T_Novel_ - T_Familiar_) / (T_Novel_ + T_Familiar_). Cisplatin-treated mice showed almost no preference for the novel object whereas those nasally administered with mitochondria performed comparable to the healthy control mice (C) The total interaction time did not differ between PBS/Vehicle and Cisplatin/Vehicle groups (n = 24-28) (D) Schematic representation of Y-maze test for assessing spatial memory. Mice were placed in one arm and their movement was monitored. (E) Perfect alternation was calculated from their sequential entry into all the arms before revisiting an arm. Cisplatin-treated mice showed decreased perfect alternations while mitochondrial administration reversed this impairment. (F) No difference was observed in the total number of arm entries between the different treatment groups (n = 18-22). Results are expressed as mean ± SEM; n = 18-28; Two-way ANOVA with Tukey's post hoc analysis *p ≤ 0.05; **p ≤ 0.01; *** p ≤ 0.001; **** p ≤ 0.0001.

To study the effect of nasally administered mitochondria on cisplatin-induced cognitive impairment, cisplatin was administered intraperitoneally at a dose of 2.3 mg/kg for five days followed by five days of rest and another five days of cisplatin as described before 17,20,21. Control mice were injected with phosphate buffered saline (PBS) following the same routine. We isolated mitochondria from human MSC and administered the organelles to the mice via the nose on the second and fourth day after the last cisplatin or PBS injection (Figure 1A). The isolated mitochondria were approximately 396 nm in size with about '87%' of the isolated fraction in this range indicating the quality of the isolated mitochondria (Figure S1). Fourteen days after the second nasal administration of mitochondria or PBS, we assessed cognitive function of the mice using the puzzle box test (PBT) for executive function, novel object place recognition test (NOPRT) and the Y-maze test of spontaneous alternation (Figures 1 and 2).

The PBT was used to evaluate executive functioning as described before (Figure 1B-F and 21). The test is based on the innate preference of mice for darkness and measures the time taken to enter the dark compartment from the bright, start compartment via an underpass. The underpass is first open (easy), then covered with bedding (intermediate), and finally covered with a lid (difficult). PBS-treated control mice learned fast to remove the lid and to enter the dark compartment while cisplatin-treated mice performed poorly or could not solve the difficult situation at all (Figure 1C-F and 21). Cumulative doses of 34, 100 and 340 µg mitochondria dose-dependently improved performance in the PBT. A significant therapeutic benefit was attained while using the cumulative dose of 340 µg, which is equivalent to mitochondria from 9x10^6^ MSC (Figure 1D-F). Nasal administration of mitochondria (170 µg) on the second and the fourth day after completion of cisplatin treatment, completely reversed the cisplatin-induced deficits in executive functioning as observed in the difficult trials of both male (Figure 1E) and female mice (Figure 1F). Spatial and working memory was tested in the NOPRT (Figure 2A-C). Mice treated with cisplatin showed a decreased preference for the novel object when compared to PBS-treated mice. Nasal delivery of mitochondria restored spatial and working memory of cisplatin-treated mice (Figure 2B). The PBS and cisplatin-treated groups did not differ in their total interaction times with the objects which denotes that the decreased discrimination index of cisplatin-treated mice was not due to reduced interest or motivation (Figure 2C). The Y-maze test was used to study spatial memory (Figure 2D-F and 21,28). The sequential exploration of all arms prior to reentering a previously visited arm was used to calculate the percentage of perfect alternations. Cisplatin treatment decreased the percentage of perfect alternations in the Y-maze, indicating impaired spatial memory. The decrease in perfect alternations after cisplatin treatment was reversed in mice subjected to nasal administration of mitochondria (Figure 2E). There were no significant differences between the treatment groups in the total arm entries (Figure 2F). Nasal administration of human mitochondria did not affect body weight (Figure S2).

### Nasally administered mitochondria arrive in the brain meninges and enter the brain parenchyma

**Figure 3:**
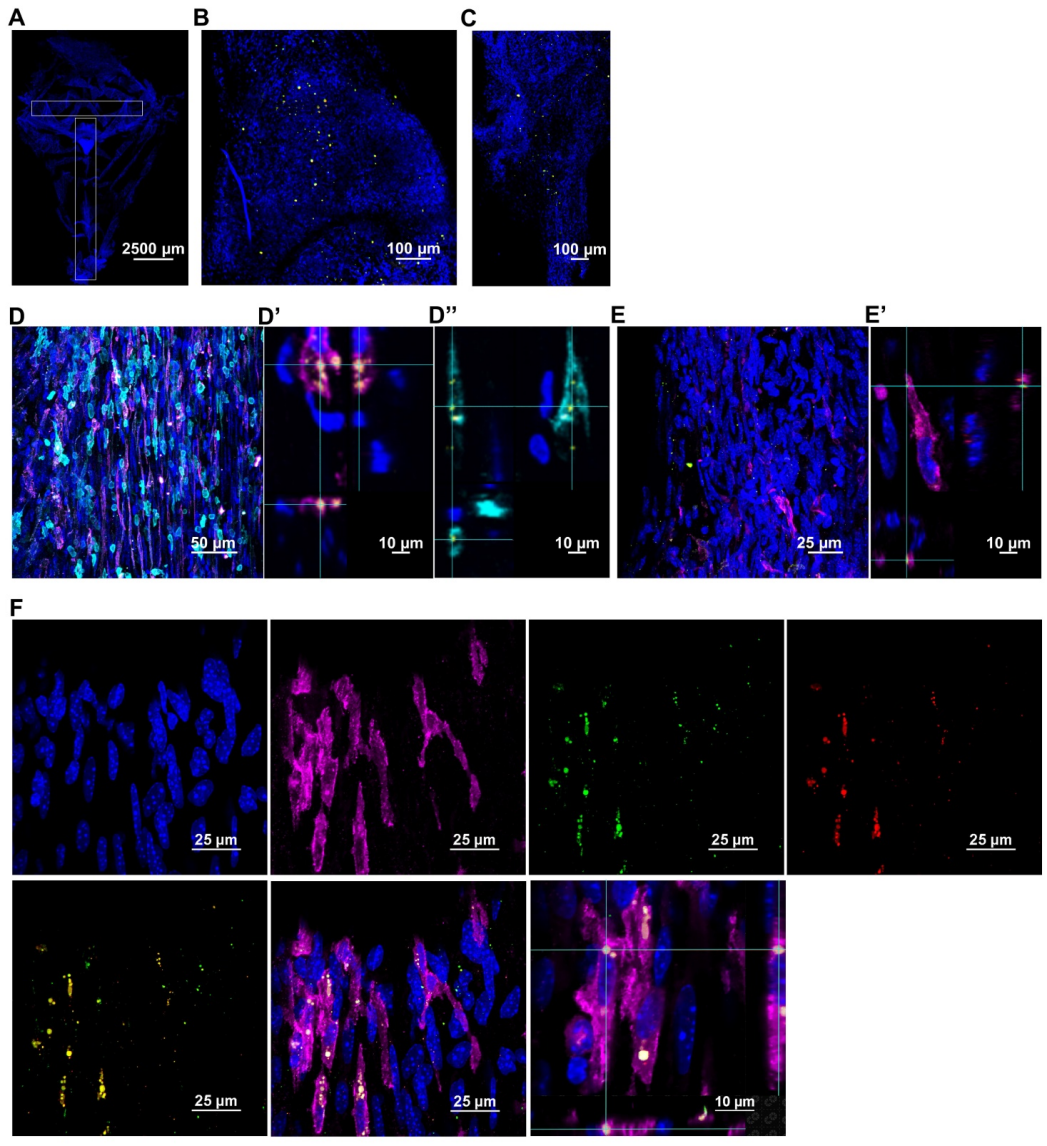
** Nasally administered mitochondria arrive in the meninges.** (A) Representation of meninges whole-mount with ROIs depicting where mitochondria were detected; scale bar 2500 µm. DsRed^+^/anti-human mitochondria^+^ mitochondria (yellow) were detected 30 min after administration near the meningeal (B) olfactory and (C) sinus regions; scale bar 100 µm. The mitochondria were in close contact with different cell types including (D) F4/80^+^ (magenta) and CD45^+^ (cyan) cells; scale bar 50 µm. 3D orthogonal slice views indicate the mitochondria are within the F4/80^+^ (magenta, D') and CD45^+^ (cyan, D'') cell bodies; DAPI^+^ nuclei appear blue. The orthogonal slice view shows the mitochondria (appearing yellow) are either well within the cell body or close to the nucleus (perinuclear); scale bar 10 µm. We did not detect human mitochondria internalized by (E) Lyve-1 (magenta) cells; scale bar 25 µm. 3D orthogonal slice view indicates the mitochondrion is outside the Lyve-1^+^ (magenta, E') cell body; scale bar 10 µm. (F) Nasally delivered DsRed^+^ (green)/anti-human mitochondria^+^ (red) mitochondria (yellow) predominantly interacted with F4/80^+^ meningeal macrophages (magenta); scale bar 25 µm and were internalized by these cells within 30 min of administration. The human mitochondria were localized within the cell body or in the perinuclear region as shown by 3D orthogonal slice view; scale bar 10 µm. Images taken with 20, 40 and 63x objectives.

Next, we explored the route taken by mitochondria isolated from human MSC stably expressing DsRed^+^ mitochondria and administered nasally 48 h after cisplatin treatment. The brain and meninges were isolated at 30 min, 3 h and 18 h post-nasal administration. The fate of the donated mitochondria was determined by immunofluorescence and high-resolution confocal microscopy. DsRed^+^ fluorescence indicates the presence of the delivered human mitochondria. Additionally, far-red quantum dots tagging an antibody directed against human mitochondria, were used to confirm the presence of the human MSC-derived mitochondria. In the whole mounts of meninges, DsRed^+^/anti-human mitochondria^+^ mitochondria were detected as early as 30 min after nasal administration and were predominantly distributed near the olfactory regions and sinuses (Figure 3A-C). Figure S3A shows that the positive signal is not due to autofluorescence because of lack of colocalization with signal in the 488 channel. Figure S3B confirms the specificity of the anti-human mitochondria antibody by showing lack of staining with an isotype control antibody. Figure S3C shows that we did not detect DsRed or anti-human mitochondria signals in cisplatin-treated mice which were not treated with mitochondria. Interestingly, the nasally applied DsRed^+^ human mitochondria were internalized by cells in the meninges (Figure 3D and F) that are positive for the leukocyte marker CD45 or the macrophage marker F4/80. We did not observe uptake of mitochondria by Lyve-1^+^ cells, a marker of meningeal lymphatic stromal cells (Figure 3E). 3-Dimensional (3D) orthogonal slice views confirmed the mitochondria were well within these CD45^+^ (Figure 3D'') or F4/80^+^ cells (Figure 3D' and F). Many of the cells containing human mitochondria were F4/80^+^ macrophages (Figure 3F). At 18 h, only a few DsRed^+^ human mitochondria were detected in the meninges (not shown).

**Figure 4:**
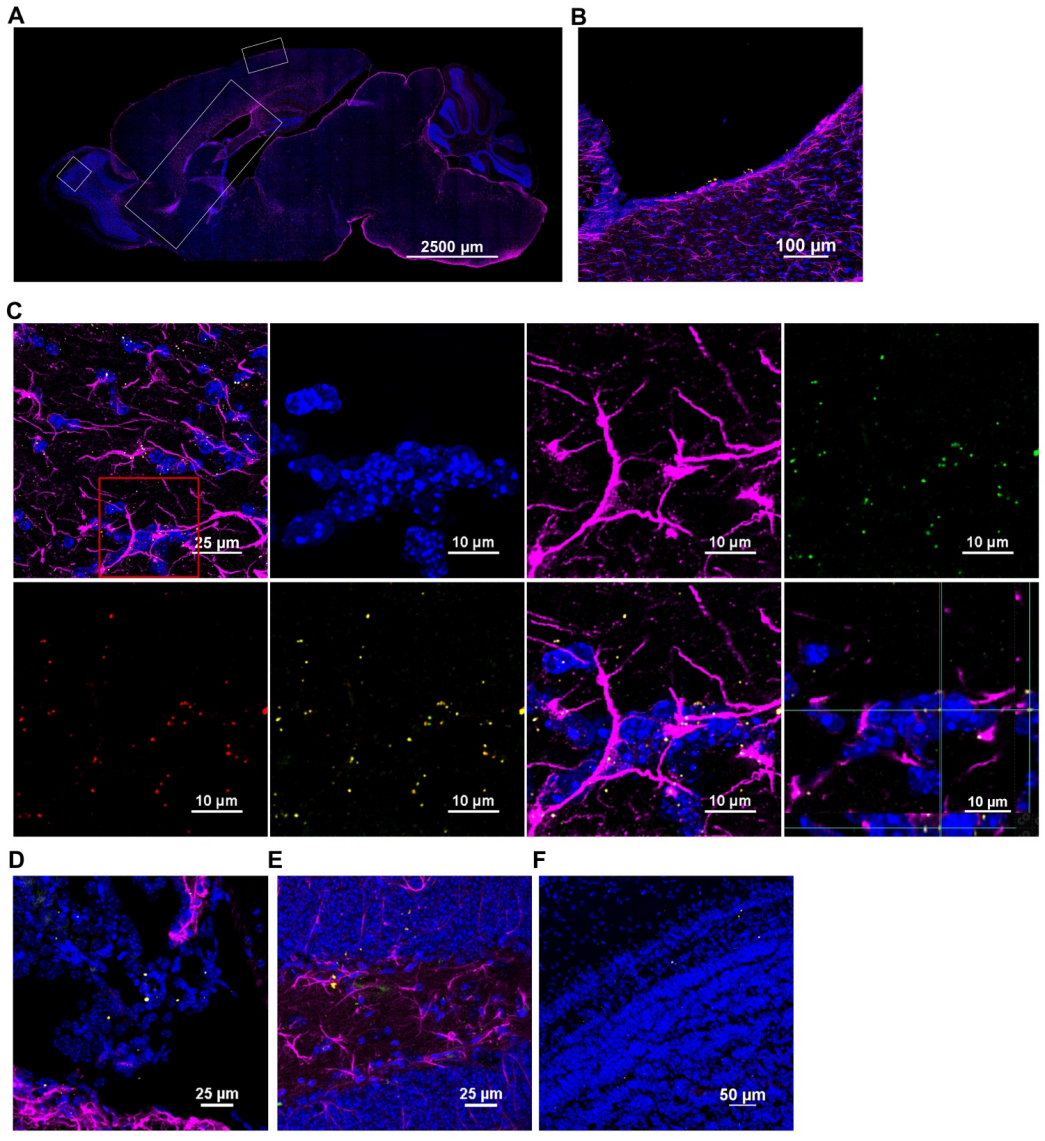
** Nasally administered mitochondria enter the brain.** (A) Representation of mouse brain sagittal section with ROIs depicting where mitochondria were detected; scale bar 2500 µm. Nasally administered DsRed^+^/anti-human mitochondria^+^ mitochondria (yellow) were detected in (B) Ventricle; scale bar 100 µm (C) RMS - DsRed^+^ (green)/anti-human mitochondria^+^ (red) mitochondria (yellow) internalized by GFAP^+^ cells (magenta) in the RMS; scale bars 25 and 10 µm (D) Choroid Plexus (E) Hippocampus (F) Olfactory bulb; scale bars 25 and 50 µm.

**Figure 5:**
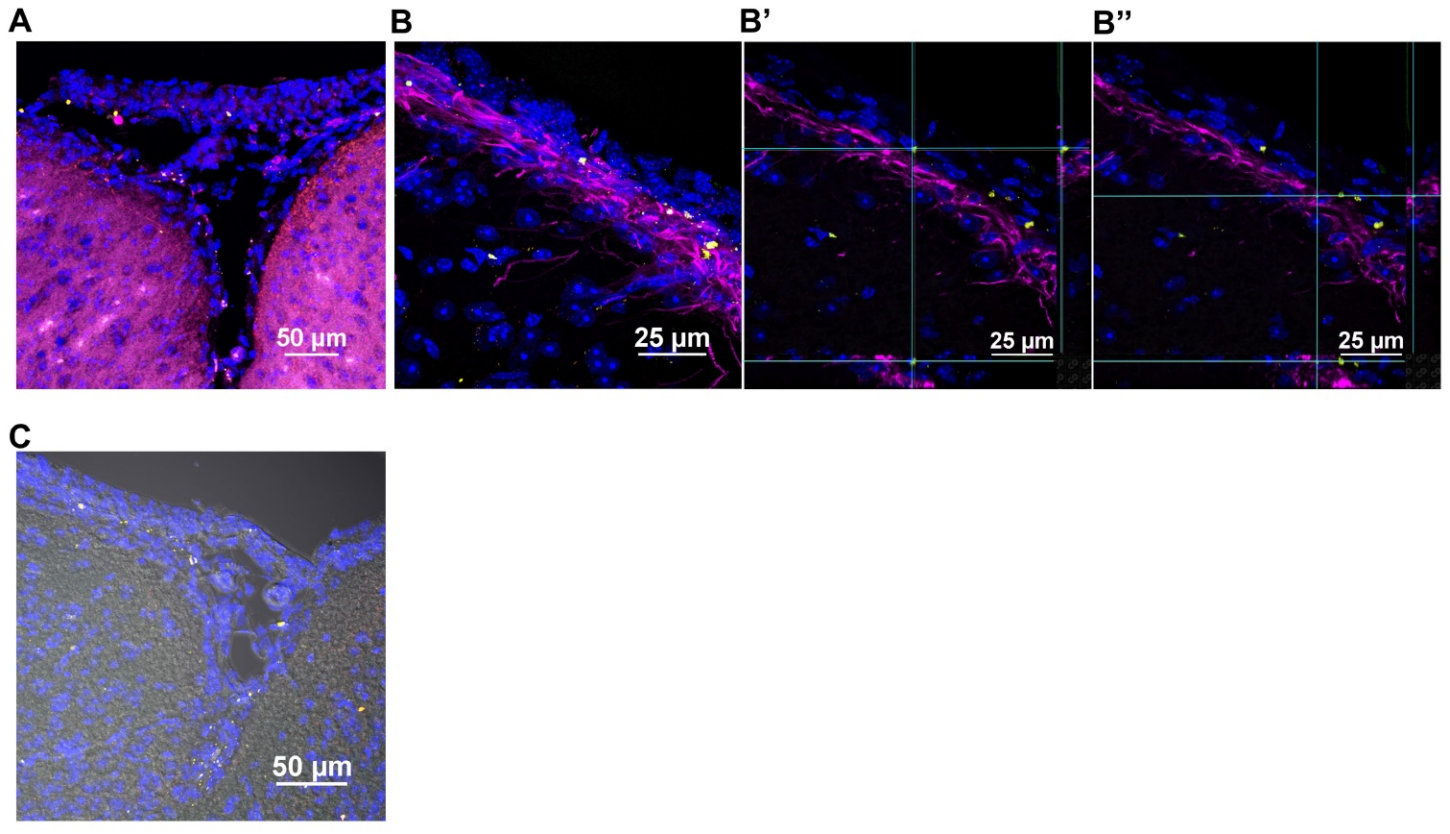
** Nasally administered mitochondria enter the pia mater and glial limitans.** (A) Pia mater with F4/80^+^ cells (magenta) adhered to the brain with administered human mitochondria (yellow); scale bar 50 µm (B) internalized by cells in the pia mater and GFAP^+^ cells in the glial limitans (magenta) as indicated by (B' and B”) 3D orthogonal slice views; scale bar 25 µm and (C) the brain parenchyma; scale bar 50 µm. Images taken with 20, 40 and 100x objectives.

Imaging similarly stained sagittal brain slices revealed that the human mitochondria gained rapid entry into the brain as well (Figure 4). At 30 min after nasal administration, a substantial number of DsRed^+^ anti-human mitochondria^+^ mitochondria were located at the ventricles (Figure 4B). By 3 h, the human DsRed^+^ mitochondria were distributed along the rostral migratory stream (RMS) (Figure 4A-C) and were internalized by glial fibrillary acidic protein (GFAP)^+^ cells (Figure 4C). The orthogonal slice views demonstrate that the human mitochondria are located well within the cells (Figure 4C). Human mitochondria were also found in the choroid plexus (Figure 4D) hippocampus (Figure 4E) and olfactory bulb (Figure 4F) at this time point. Additionally, the administered mitochondria were seen entering the brain from the pia mater - closely surrounding the brain parenchyma, where they were present at 30 min and even 18 h after administration, interacting with GFAP^+^ cells (Figure 5A-C). 3D orthogonal slice views confirm that the human mitochondria are internalized by cells in the pia mater (Figure 5B') and glial limitans (Figure 5B''). The results in Figure S5A-B show that the DsRed signal also colocalized with an antibody recognizing human mitochondrial transcription factor A (mtTFA). Negative controls depicted in Figures S4C and S5C demonstrate that we did not detect DsRed, anti-human mitochondria or anti-human mtTFA signal in samples from mice that were treated with cisplatin but did not receive the human mitochondria. In addition, samples stained with an IgG control antibody were negative (Figure S4A-B). Together these data confirm specificity of the signal for human mitochondria.

### Nasally delivered mitochondria repair cisplatin-induced loss of white matter integrity

**Figure 6:**
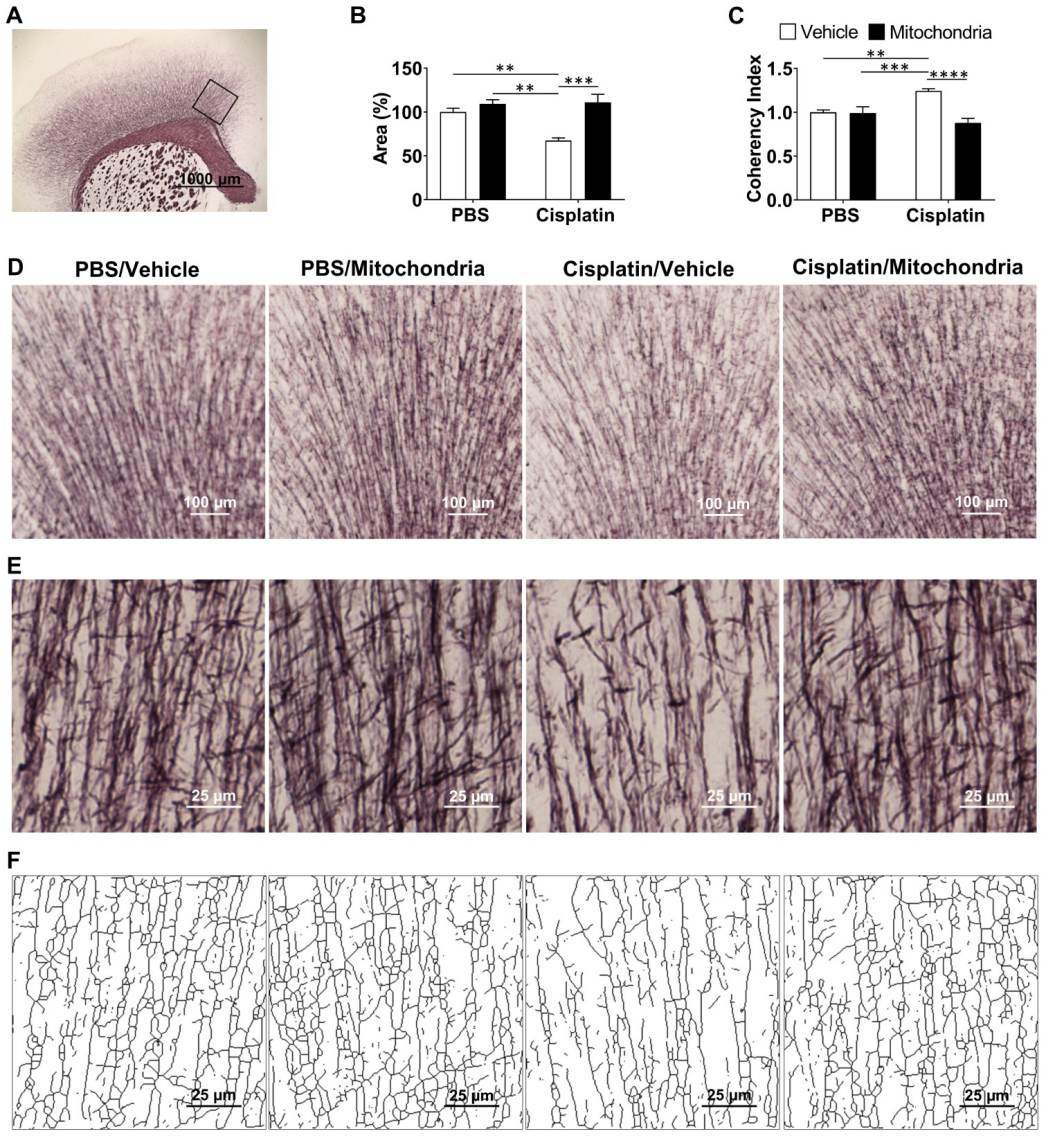
** Nasally administered mitochondria repair cisplatin-damaged white matter integrity.** (A) Mouse brain coronal section stained with Black Gold II with ROI indicating the cingulate cortex where white matter integrity was analysed; scale bar 1000 µm. Cisplatin-treatment (B) reduced percent area stained with Black Gold II and (C) increased coherency index which inversely relates to the complexity of myelin organization. Nasal administration of mitochondria reversed this damage. (D) Low magnification (4x) images representing Black Gold II^+^ area; scale bar 100 µm. (E) Higher magnification images (20x) representing the myelin organization; scale bar 25 µm. (F) Skeletonization of these higher magnification images revealed the complexity of the myelin arborization; scale bar 25 µm. Results are expressed as mean ± SEM; n = 4-8; Two-way ANOVA with Tukey's post hoc analysis **p ≤ 0.01; *** p ≤ 0.001; **** p ≤ 0.0001.

Previously, we have shown that cisplatin treatment results in structural damage to the white matter, especially in the cingulate cortex, as visualized using myelin basic protein and Black Gold II staining approaches 20,21. To evaluate the effect of nasally delivered mitochondria on white matter integrity, Black Gold II myelin staining of the mouse brain was performed (Figure 6). We confirm here that cisplatin treatment significantly decreases the Black Gold II^+^ area in the cingulate cortex (Figure 6A-B, D and 31). The complexity or arborization of myelin organization is measured inversely as coherency. Cisplatin treatment also increased the coherency of myelin fibers in the cingulate cortex as compared to PBS control mice (Figure 6C, E-F and 31). Nasal administration of mitochondria reversed the loss of Black Gold II-positive area and normalized fiber coherency, demonstrating that the nasally delivered mitochondria reversed the cisplatin-induced structural myelin damage (Figure 6A-F).

### Nasally administered mitochondria resolve cisplatin-induced synaptic damage

**Figure 7:**
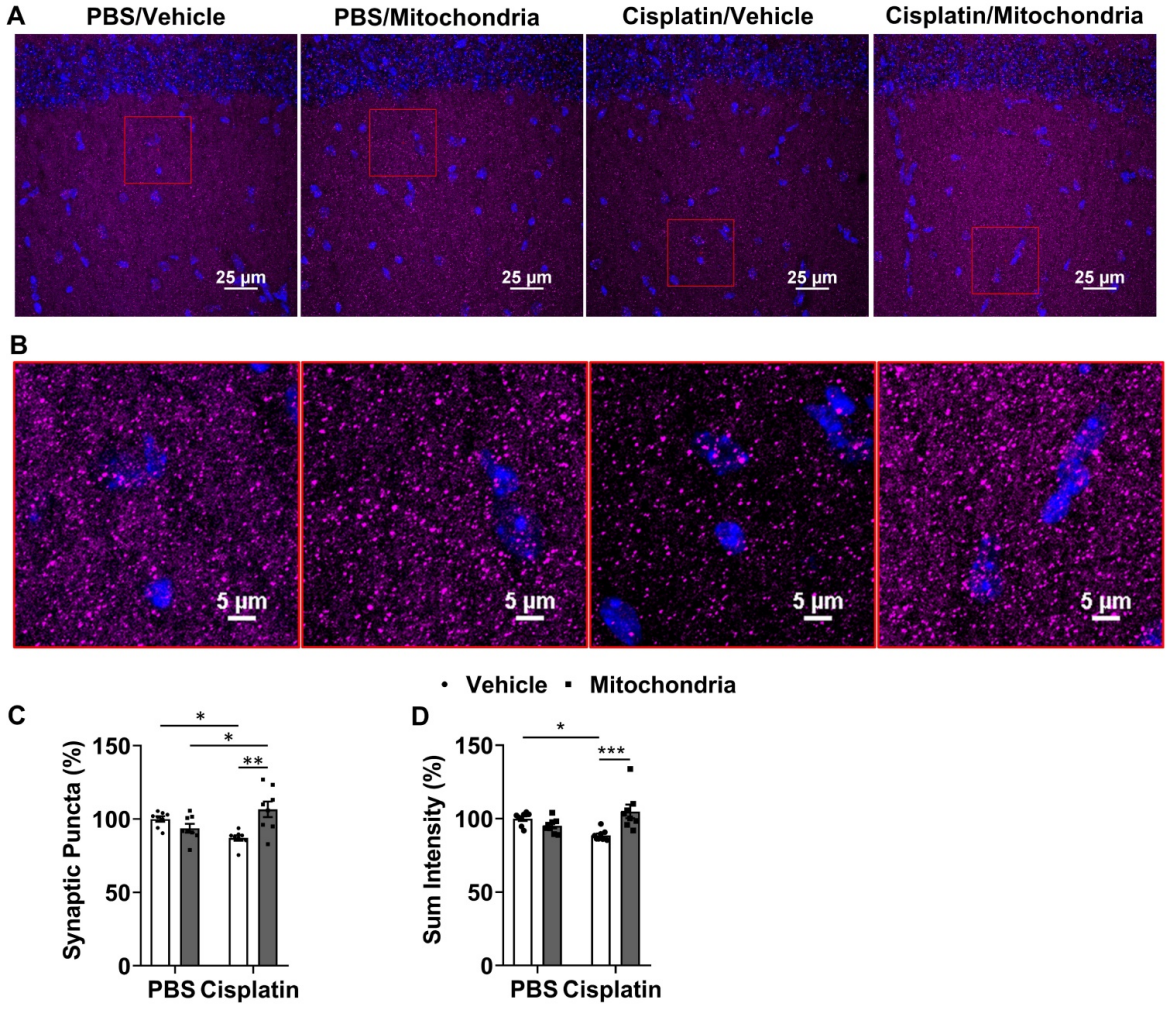
** Nasally administered mitochondria reverse cisplatin-induced synaptic loss.** (A) Mice hippocampal regions stained with synaptophysin for different treatment groups; scale bar 25 µm. ROI indicates (B) clear synaptophysin^+^ synaptic puncta at higher magnification; scale bar 5 µm. Cisplatin-treated mice showed reduction in (C) percentage of synaptic puncta and (D) sum intensity while nasal administration of mitochondria reversed this loss. Results are expressed as mean ± SEM; n = 8; Two-way ANOVA with Tukey's post hoc analysis *p ≤ 0.05; **p ≤ 0.01; *** p ≤ 0.001.

To evaluate the effect of nasally delivered mitochondria on synapses, we used the pre-synaptic marker synaptophysin and quantified its expression in the hippocampus. The number of synaptophysin^+^ punctate structures and total fluorescence intensity were measured. Cisplatin treatment decreased the number of synaptic puncta and total intensity. Both signs of loss of pre-synaptic integrity were reversed by nasal administration of mitochondria to cisplatin-treated mice (Figure 7A-D).

### Nasally delivered mitochondria reverse cisplatin-induced mitochondrial defects and compromised-membrane integrity in synaptosomes

**Figure 8:**
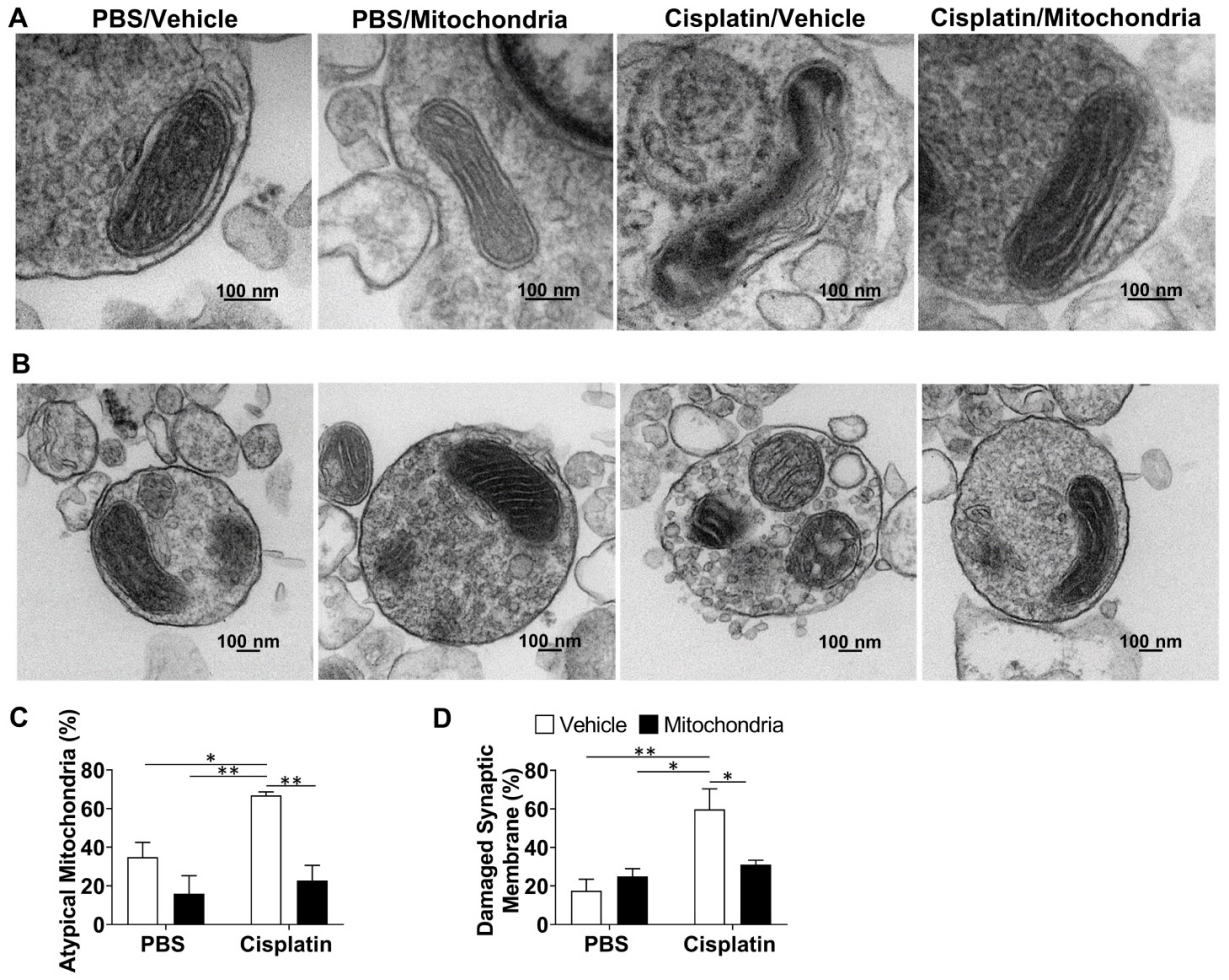
** Nasally administered mitochondria resolve cisplatin-induced synaptosomal mitochondrial defects and membrane integrity.** (A) Representative TEM images depicting the ultrastructure of mitochondria in brain synaptosomes for different treatment groups. Mitochondrial swelling, membrane ruffling and cristae disorganization indicate defective or atypical mitochondria; scale bar 100 nm. (B) Representative TEM images showing whole synaptosomes. Membrane ruffling and disruption, blebs and vesicle leakage were considered to indicate compromised membrane integrity; scale bar 100 nm. Synaptosomes of cisplatin-treated mice revealed (C) high percentage of atypical mitochondria and (D) high percentage of damaged synaptic membrane. Results are expressed as mean ± SEM; n = 4; Two-way ANOVA with Tukey's post hoc analysis *p ≤ 0.05; **p ≤ 0.01.

We have previously proposed that neuronal mitochondrial abnormalities underlie the cognitive impairment and associated brain damage as a result of cisplatin treatment 21. To investigate if the nasally delivered mitochondria repair the defective mitochondria, we isolated brain synaptosomes and analysed their mitochondrial structure by transmission electron microscopy (TEM). The mitochondria present in the synaptosomes of cisplatin-treated mice displayed swelling, membrane ruffling and cristae disorganization, which are typical characteristics of dysfunctional mitochondria. Nasal delivery of mitochondria reversed these mitochondrial abnormalities in the whole brain synaptosomes of cisplatin-treated mice (Figure 8A and C)*.* We noticed that the synaptic membranes of synaptosomes from cisplatin-treated mice were particularly ruffled, disrupted, leaky and with blebs (Figures 8B and S6). Nasal administration of mitochondria normalized the integrity of the synaptosomes (Figure 8B and D).

### Nasally delivered mitochondria induce transcriptomic alterations in the hippocampus

**Figure 9:**
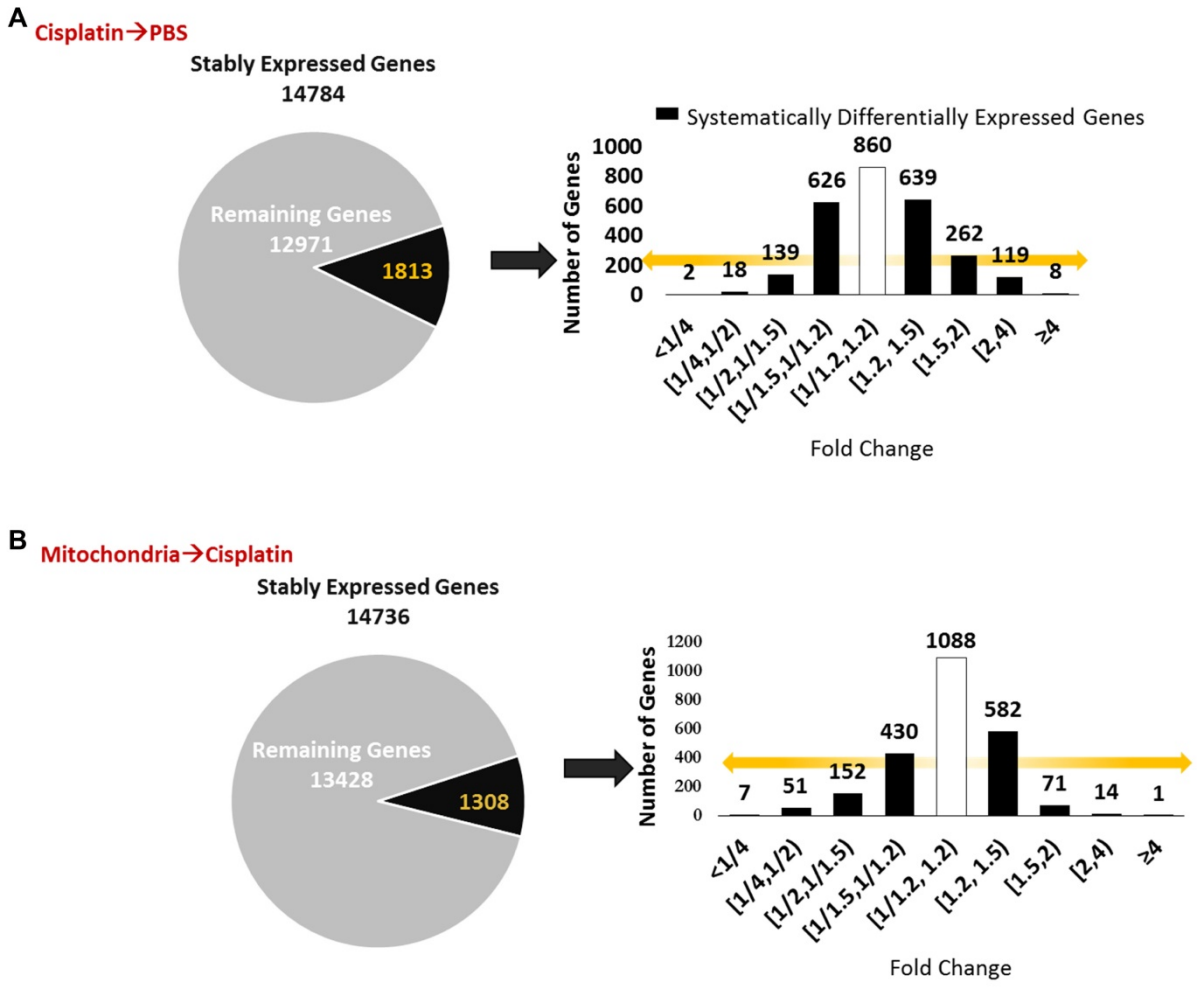
** Nasally delivered mitochondria induce transcriptomic changes in the hippocampus, the brain region crucial for cognition.** RNA-sequencing was performed on hippocampi collected 72 h after the last administration of mitochondria. (A) Cisplatin treatment revealed 14874 stably expressed genes of which 1813 genes were systematically differentially expressed with fold change < 1/1.2 and > 1.2. (B) Nasal administration of mitochondria to cisplatin-treated mice revealed 14736 stably expressed genes of which 1308 were systematically differentially expressed. n = 4.

**Figure 10:**
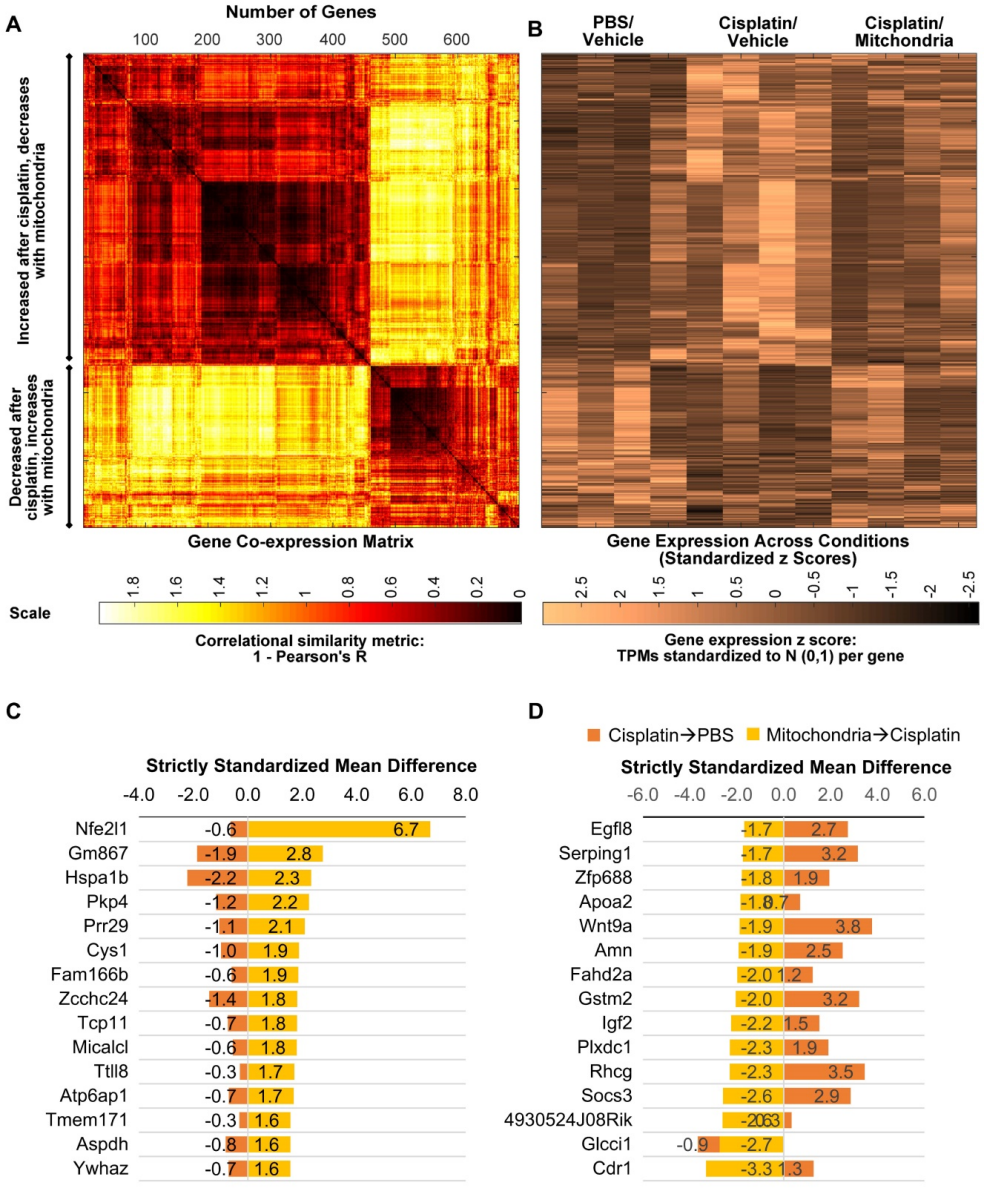
** Nasally delivered mitochondria reverse cisplatin-altered genes in the hippocampus.** RNA-sequencing was performed on hippocampi collected 72 h after the last administration of mitochondria. (A) Nasal administration of mitochondria reversed 698 genes altered by cisplatin-treatment in the hippocampus. Co-expression matrix shows subsets of genes decreased by cisplatin-treatment and increased by mitochondria as well as those increased by cisplatin and decreased by mitochondria. (B) Heat map of gene expression based on standardized z scores reveal expression pattern across different treatment conditions. Top 15 correlating genes based on strictly standardized mean difference (C) downregulated by cisplatin and upregulated by mitochondria and (D) upregulated by cisplatin and downregulated by mitochondria. n = 4.

We next explored whether the regenerative effect of nasal mitochondrial donation was associated with a change in the transcriptome of the hippocampus, an area crucial for cognition. The hippocampi were collected and processed for RNA-Sequencing 72 h after the second dose of mitochondria. We selected this time point because we had observed in an earlier study that the regenerative effect of MSC was clearly present in the hippocampus 72 h after the last nasal administration 21. We compared the hippocampi of PBS-treated mice versus cisplatin-treated mice; and cisplatin-treated mice with and without mitochondrial donation. Approximately 16000 coding gene abundances (transcripts per million, TPM > 0.0) were quantified for each sample. Among these, genes with TPM > = 0.5 were considered stably expressed (Supplementary File 1, Sheet 1 and Table S1). Stably expressed genes with strictly standardized mean difference (SSMD) scores of > = 0.75 or < = -0.75 were identified as systematically changing genes. For these systematically changing, stably expressed genes in both comparisons, we counted the number of genes that had fold change in the half-open or open intervals defined by { 0, 1:4, 1:2, 1:1.5, 1:1.2, 1.2:1, 1.5:1, 2:1, 4:1, +Infinity } (Figure 9A-B). 14784 genes were found to be stably expressed in mice treated with cisplatin followed by PBS administration. Of these, 1813 genes were systematically differentially expressed displaying a fold change ≥ 1.2 or ≤ 1/1.2 when compared to PBS-treated mice (Figure 9A). Amongst the 14736 genes stably expressed in mice treated with mitochondria after cisplatin treatment, 1308 genes were differentially expressed with a fold change ≥ 1.2 or ≤ 1/1.2 as compared to cisplatin-treated mice (Figure 9B). Stably expressed, systematically changing genes with a fold change > = 1.5:1 or < = 1:1.5 in either comparison are shown in Supplementary File 1, Sheet 2. Among these genes, for genes which were stably expressed in at least one condition in both the comparisons, the SSMD vectors for each comparison were compared by calculating Pearson's Correlation and the corresponding p-value (Supplementary File 1, Sheet 3). Administration of mitochondria to cisplatin-treated mice reversed the cisplatin-induced change in expression of 676 genes as represented in the co-expression matrix (Figure 10A) and heat map (Figure 10B). The expression pattern in the heat map shows a certain degree of variability among the replicates within the same treatment group indicating the plasticity of the hippocampus (Figure 10B). Top anti-correlated genes include *Nfe2l1, Atp6qp1 and Apoa2* (Figure 10C-D). *Nfe2l1* or Nuclear Factor Erythroid 2-Related Factor 1 has been reported to promote neuronal protection from stress-induced apoptosis and to be involved in the regulation of antioxidant mechanisms 32. Loss of *Nfe2l1* is associated with neurodegeneration 33. In our dataset, *Nfe2l1* expressed a SSMD of 6.7 induced by the administration of mitochondria to cisplatin-treated mice (Figure 10C).

**Figure 11:**
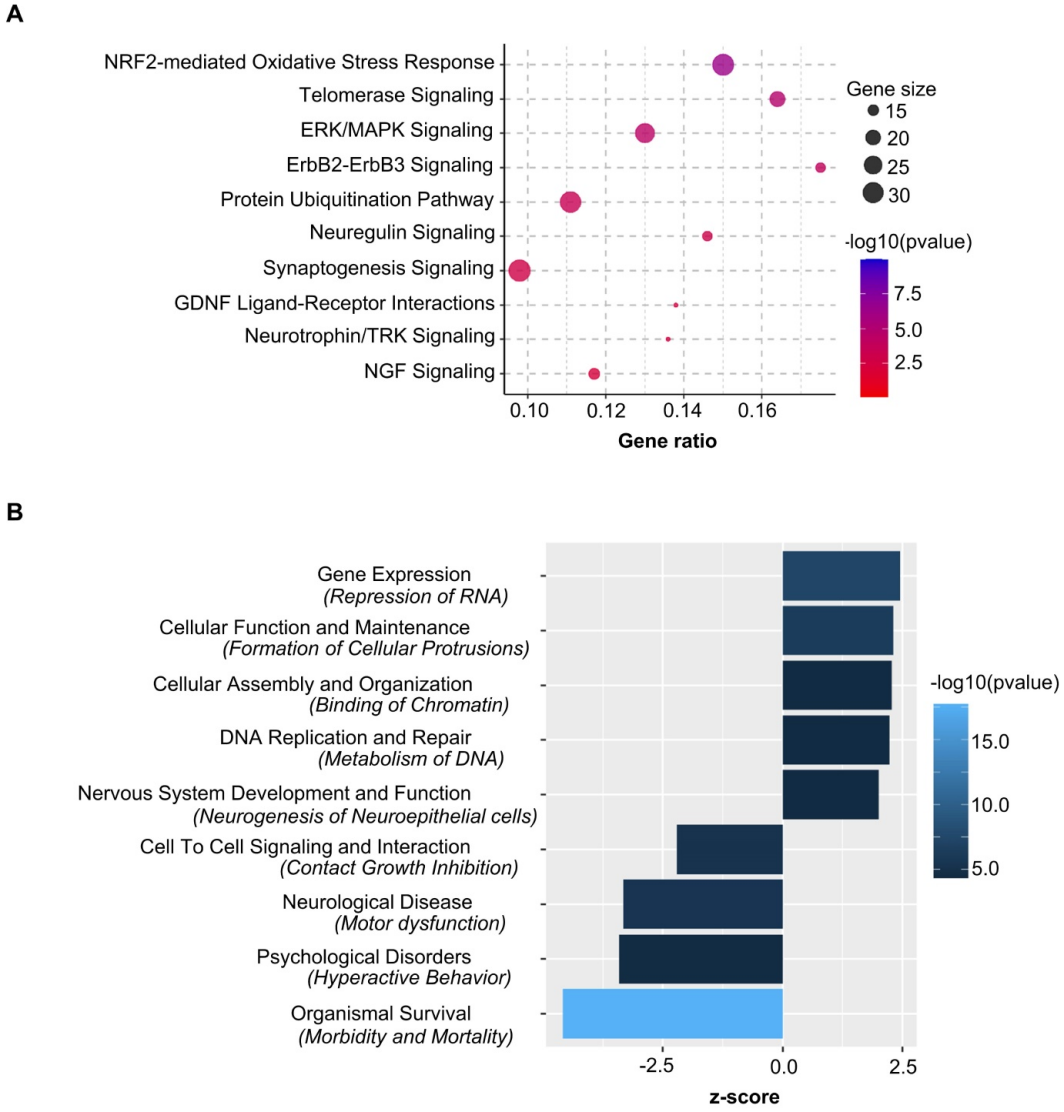
** Nasally delivered mitochondria activate key canonical pathways and regulate crucial functions in restoring cisplatin-induced cognitive deficits.** Ingenuity Pathway Analysis of stably expressed genes revealed (A) top relevant canonical pathways activated and (B) crucial functions regulated by the nasal administration of mitochondria in cisplatin-treated mice. n = 4.

Ingenuity Pathway Analysis showed *Nrf2*-mediated oxidative stress response as the top canonical pathway along with Telomerase, ERK/MAPK and Synaptogenesis Signaling regulated by the nasal administration of mitochondria to cisplatin-treated mice (Figure 11A). Pathway prediction suggests that the activation of *Nrf2*-mediated response may regulate antioxidant proteins towards minimizing oxidative damage. Protein repair and clearance may also be triggered by ubiquitination, proteosomal degradation and regulation of chaperone and stress response proteins (Figure S7). Pathway analysis also predicted several vital functions regulated by nasally administering mitochondria to cisplatin-treated mice (Figure 11B and Table S2). Analysis of upstream regulators that could underlie the change in expression in response to administration of mitochondria to cisplatin-treated mice revealed 33 molecules with a z-score ≥ 2 that were predicted to be activated and 27 molecules with a z-score ≤ -2 that were predicted to be inhibited (Table S3). Rictor, Ngf, Cd40, Hsf1, Il4, Adora2a and Fgf2 were some molecules that were predicted to be activated while Sparc, Cd44, Il10ra and 5 other micro-RNAs were among those that were predicted to be inhibited. One of the upstream regulators that was predicted to be activated (z-score of 2.93) in response to administration of mitochondria to cisplatin-treated mice is the regulatory protein Rictor, which is a sub-component of the mammalian target of rapamycin complex 2 (mTORC2). Interestingly mTORC2 localizes to the mitochondria-associated endoplasmic reticular membrane and mTORC2-deficiency disrupts mitochondrial function including ATP production and calcium uptake 34. Comparative pathway analysis between administration of mitochondria to cisplatin-treated and PBS mice revealed that the transcriptome regulation by mitochondria was specific to cisplatin-treatment and not merely an effect which would also occur in healthy mice (data not shown).

## Discussion

Our findings demonstrate that mitochondria isolated from human MSC and delivered via the nasal route, resolve chemotherapy-induced cognitive impairment and its associated pathologies such as white matter and synaptic loss as well as the underlying synaptosomal mitochondrial deficiencies, in a mouse model. For the first time, we demonstrate the therapeutic effects of only two administrations of mitochondria which, when delivered nasally, completely restored within two weeks the executive functioning, spatial recognition and working memory impaired by cisplatin treatment (Figures 1 and 2). We also show for the first time that within 30 min of nasal administration, the mitochondria were detectable in the meninges where they were predominantly internalized by macrophages (Figure 3). Within this 30 min, the mitochondria also arrived at the ventricles and choroid plexus, gaining access to the brain. 3 h after delivery, they were found distributed along the RMS where they were internalized by GFAP^+^ cells. By this time the nasally administered mitochondria also reached the hippocampus (Figure 4). 3D orthogonal slice analysis indicated that the mitochondria were internalized by the F4/80^+^ or GFAP^+^ cells within a short span of nasal administration (Figures 3,4 and 5) and not freely or randomly distributed in an extracellular environment. Evidently, these mitochondria that had entered the meninges and brain completely restored the cisplatin-induced white matter damage in the cingulate cortex (Figure 6), synaptic loss in the hippocampus (Figure 7), and the compromised synaptosome membrane integrity and structural mitochondrial defects in synaptosomes (Figure 8) as observed 35 days after the second mitochondrial donation. Furthermore, our study is the first to report that the nasally delivered mitochondria regulate the hippocampal transcriptome within 72 h after the second administration (Figures 9, 10 and 11).

In our mouse model of cisplatin-induced cognitive impairment, we used a cumulative dose of 23 mg/kg which inhibits tumor growth as well 21. This dose corresponds to a Human Equivalent Dose of 70 mg/m^2^ 35 which is well within the 50 to 100 mg/m^2^ range that is given to patients 36. In a recent study, we demonstrated that nasal administration of human or murine intact MSC could reverse cisplatin-induced cognitive impairment in mice 21. In another recent study we showed that MSC donate their healthy mitochondria to cisplatin-damaged neural stem cells (NSC) leading to restoration of their mitochondrial membrane potential and improved survival 37. These collective in* vivo* and *in vitro* findings led us to propose that, in lieu of administering whole intact MSC, isolated mitochondria can be used to resolve cisplatin-induced cognitive deficits and associated structural and functional changes. Previously, we showed that mouse MSC were effective as human MSC in reversing cisplatin-induced cognitive deficits, in mice. Here, we used mitochondria isolated from human MSC in our mouse model for the primary purpose of being able to trace the administered mitochondria with a human-specific mitochondrial antibody. We did not notice any adverse reaction of the mice to the xenogeneic mitochondria. One topic of concern usually raised with the administration of isolated mitochondria is the possibility that they may function as damage-associated molecular proteins (DAMPs) which could lead to (neuro)inflammation 38. However, we did not see activation of inflammatory pathways in the hippocampal transcriptome using human mitochondria at the cumulative dose of 340 µg. Mitochondria lack many surface antigens including HLA-Class 1 antigens thereby exhibiting lower immunogenicity than MSC. This is one advantage favoring the clinical translation of allogeneic donation of mitochondria over MSC 39. While MSC can be easily expanded for clinical use, the risk always remains that the cells may integrate into the system such as the brain, lymph nodes or bone marrow with a possibility of inducing or enhancing tumor formation 40-42. Use of isolated mitochondria circumvents this problem and we therefore propose that mitochondrial donation may be a safer therapeutic approach for cancer and cancer treatment-induced neurotoxicities. Notably, the concept of mitochondrial administration is also being considered the treatment of Parkinson's disease 43, cerebral 44 and cardiac ischemia 45, cancer 46, diabetic nephropathy 47 and spinal cord injuries 48,49 and some clinical trials for evaluating the safety and efficacy of isolated mitochondria based therapies have also recently commenced.

An important benefit of our approach is the nasal route of delivery. In case of central nervous system (CNS)-targeted delivery of therapeutics, intracranial administration poses serious risk of injury. Intravenous administration has the disadvantage that it may lead to accumulation in the lung 50 and liver 51 requiring high doses which may generate inflammatory reactions 52 or other adverse effects 53. Systemic administration of therapeutics intended to reach the brain are also obstructed by the blood-brain and blood-cerebrospinal fluid (CSF) barriers. In contrast, the nasal route of delivery is simple, non-invasive and facilitates the delivery of therapeutics to the brain thereby reducing the amount of mitochondria required.

This is the first report of nasally delivered cellular organelles of about 396 nm (Figure S1) in size to be detected in the meninges (Figure 3). The mitochondria in the meninges were detected inside cells, particularly F4/80^+^ macrophages (Figure 3D and F). Uptake of MSC-derived mitochondria by macrophages may lead to metabolic reprogramming to an anti-inflammatory repair phenotype 54 which may contribute to restoration of brain function and structure. Interestingly, we detected the human mitochondria in the pia mater - the innermost meningeal layer closely attached to the brain and in the brain parenchyma as well (Figure 5). This suggests that donated mitochondria can also directly act in neurons and glia. It is to be noted that the isolated mitochondria have a tendency to adhere to each other and hence appear micron-sized.

Multiple previous studies have shown that nasally administered MSC enter the brain parenchyma of mice or rats with brain damage caused by cisplatin, hypoxic ischemic insults or in models of neurodegenerative disorders 21,55-58. The entry of MSC into the brain is facilitated by pretreatment of the nasal cavity with hyaluronidase, an approach we used here as well 59. Studies in the 1930s already showed that nasally administered bacteria can cross the nasal epithelium within minutes, indicating a paracellular route of entry 60,61. One of the main mechanisms by which nasally delivered therapeutics reach the brain is by crossing the nasal epithelium via a paracellular route after which they can enter the CSF via the perineurial space 55. Through the CSF, the mitochondria can reach the cells of the arachnoid membrane (middle layer) and pia mater of the meninges. From the subarachnoid space, the mitochondria may reach the ventricles of the brain from where they can enter the RMS, hippocampus, and other brain regions as well as the periphery. Indeed, we detected nasally delivered mitochondria in the olfactory bulb and the RMS where they can interact with astrocytes and NSC as we detected human mitochondria in GFAP^+^ cells.

It has been shown that pro-inflammatory cytokines can enhance the transfer of mitochondria from MSC to cardiomyocytes, airway epithelial cells, and retinal ganglion cells 62-64. However, we reported previously that we do not detect signs of inflammation in the brain of cisplatin-treated mice. Specifically, we did not detect changes in expression of the prototypic pro-inflammatory cytokines IL1β, IL6, and TNFα, or of the markers of glial activation GFAP and CD11b as measured by real time RT-PCR analysis in the hippocampus and prefrontal cortex 20. In addition, we previously performed RNA-sequencing analysis of the effect of cisplatin on the gene expression profile in the hippocampus and in the sensorimotor cortex of cisplatin-treated mice 21,65. The results of these analysis confirmed that there is no evidence for activation of inflammatory pathways in the brain. Consistently, the RNA-sequencing data included here do not identify changes in inflammatory pathways in the hippocampus of cisplatin-treated mice. Thus, it is unlikely that inflammation contributed to the uptake of nasally administered human mitochondria in the present study. Mitochondrial transfer to damaged cells including cisplatin-treated neuronal cells, has been shown to result in an increase in oxidative metabolism and improved cellular function including an anti-inflammatory phenotype in macrophages 30,37,54. We propose that nasally delivered mitochondria may repair the acceptor cells like neurons, macrophages, and GFAP^+^ cells possibly by changing their metabolic programming towards restoration of the damage and/or a more restorative phenotype.

In a previous study, RNA-sequencing of the hippocampus 72 h after the second nasal MSC administration demonstrated changes in expression of genes involved in the top canonical pathways of oxidative phosphorylation and mitochondrial dysfunction 21. Although nasal delivery of mitochondria or MSC both restored mitochondrial defects (Figure 8 and 21), the alterations induced by the mitochondria in the hippocampal transcriptome were different from those induced by MSC at the same time point. The top canonical pathways activated as a result of nasal administration of mitochondria to cisplatin-treated mice included the *Nrf2*-mediated oxidative stress response and telomerase signaling, ERK/MAPK signaling and synaptogenesis signaling (Figure 11A). Recently, *Nrf2* ablation was shown to promote Alzheimer's Disease-like pathology in mice highlighting the therapeutic role of *Nrf2* pathway in resolving cerebral deficits 66,67. In accordance with the transcriptomic analysis, we observed the restoration of hippocampal synaptic loss and repair of synaptic membrane integrity (Figures 7 and 8). Pathway analysis predicts the activation of the *Nrf2*-mediated response may result in regulation of antioxidant proteins to reduce oxidative damage. It also predicts that ubiquitination and proteosomal degradation along with regulation of chaperone and stress response proteins may lead to protein repair and clearance (Figure S7).

Furthermore, 676 genes which had been altered by cisplatin, showed a reversal of that change in response to administration of mitochondria (Figure 10). Among these anti-correlative genes, the *Nfe2l1* gene was upregulated upon administration of mitochondria to cisplatin-treated mice (Figure 10C). Notably, the nuclear factor, erythroid 2 like 1 (Nfe2l1) protein promotes cellular protection against oxidative stress by triggering antioxidant genes through the glutathione synthesis pathway 68. Selective deletion of the *Nfe2l1* gene in the neuronal cells of Camk2Cre;Nrf1^-/flox^ mice has resulted in impaired function of proteasomes, establishing Nfe2l1 as a key transcriptional regulator of neuronal proteasomes and its involvement in neurodegenerative pathogenesis 33. Taken together, we predict on the basis of RNA-sequencing data that nasally administered mitochondria reverse cognitive deficits and promote brain health involving activation of the *Nrf2* pathway. Together, these results suggest that nasally delivered mitochondria trigger significant changes in the hippocampal transcriptome within 72 h of administration contributing to the restoration of the oxidative-anti-oxidative balance ensuring repair of cisplatin-induced brain damage.

With the advent of clinical trials using isolated mitochondria, questions are raised on the ability of isolated mitochondria to survive in high extracellular calcium levels upon administration and to generate sufficient energy to enter cells and continue to function after cellular uptake 69-72. Transfer of exogenous mitochondria into damaged cells or tissues have shown maintenance or improvement of cellular bioenergetics in the injured recipient cells in the case of spinal cord injury and cardiac ischemia 48,73. Administered mitochondria were quickly internalized by the ischemic cardiac cells *in vitro* and *in vivo* with calcium levels 1.7 and 1.8 mM, respectively. Upon internalization, mitochondrial donation enhanced the bioenergetics of the damaged cells for at least 21 days 73. In our study, we maintained the mitochondria in calcium-free mitochondrial respiration buffer until ready for nasal delivery when they were transferred to calcium-free PBS. Upon administration, the mitochondria are probably subject to calcium levels of about 0.5 mM in the olfactory mucus 74 and about 1.2 mM in the CSF 75. Upon reaching the meninges, the mitochondria were rapidly internalized by the meningeal cells (Figure 3). In the brain they were also internalized within a short span (Figures 4 and 5) indicating they were not exposed to a high calcium extracellular environment for prolonged duration.

It is still unknown how donated mitochondria repair damaged neuronal cells. Interactions between mitochondrial and nuclear genes are vital for fundamental cellular processes such as respiration, transcription and translation 76,77. We observed that the internalized mitochondria, in many instances were localized close to the nucleus (perinuclear), as if communicating with them (Figures 3 and 4). In the literature it has been suggested that donated mitochondria can fuse with mitochondria of damaged recipient cells and thereby repair the bioenergetic machinery 78 or replenish mitochondrial DNA in the acceptor cell 79. Although this might be true, it remains difficult to accept that the few MSC-derived mitochondria can restore cellular respiration by physical fusion to the many acceptor cell mitochondria. Many signaling routes between the mitochondria and nucleus have evolved. It is more likely that the donated mitochondria activate a transcriptional program leading to enhanced Nrf2 signaling and subsequent recovery of host mitochondrial function by a host of antioxidants 80. In this respect it will be of interest to investigate whether nasal application of the donated mitochondria just provide the timely and necessary 'danger signals' to the nucleus in a more efficient way than the long-term damaged mitochondria from the acceptor cell can do as a result of cisplatin treatment. Identification of the mode of presentation of the activating mitochondrial danger signals by the donated mitochondria could provide an intriguing insight into mitochondrial repair and brain health.

Our findings elucidate the therapeutic effects of nasally delivered mitochondria to resolve unmet needs in the treatment of cancer survivors with neurotoxicities and highlights its potential significance for clinical translation. It also provides promise for treatment of a range of cognitive and neuronal deficits warranting further investigation in large animal models.

## Materials and Methods

### Animals

C57BL/6J mice, 8 weeks of age (Jackson Laboratory) were housed at constant temperature of 22 ± 2 ˚C and 12/12 h in reverse dark-light cycle (dark 830-2030 h) with food and water *ad lib*. Mice were randomly assigned to the treatment groups. All animal experiments were performed at The University of Texas Health MD Anderson Cancer Center, Houston, Texas according to procedures approved by the Institutional Animal Care and Use Committee.

### Isolation of Mitochondria from Human MSC

Human MSC lentivirally transduced with mitochondria-targeted *PDHA1-DsRed* to express *PDHA1-DsRed* mitochondria were provided by Dr. Rodrigo Jacamo of the University of Texas MD Anderson Cancer Center, Houston, Texas, USA. The human MSC were cultured in Minimal Eagle Essential Medium (Corning Life Sciences, Corning, NY, USA) supplemented with GlutaMAX^TM^ (ThermoFisher Scientific, Gibco, Life Technologies Corporation, Grand Island, NY, USA), 2 USP units/mL heparin (Fresenius Kabi USA, Lake Zurich, IL), 7.5% heat inactivated Fetal Bovine Serum (FBS, Sigma-Aldrich, St. Louis, MO, USA), 2.5% platelet lysate (PLTMax^®^, Mill Creek Life Sciences, Rochester, MN, USA) and 1% penicillin-streptomycin (Sigma-Aldrich, St. Louis, MO, USA).

Mitochondria were isolated using the Mitochondria Isolation Kit for Cultured Cells (ThermoScientific, Pierce Biotechnology, Rockford, IL, USA) following the kit protocol - Dounce homogenization method and maintained in respiration buffer 45. Immediately prior to nasal administration, the mitochondria were pelleted and resuspended in PBS.

### Cisplatin Treatment and Nasal Administration of Mitochondria

To induce cognitive impairment, mice were injected with PBS or cisplatin in PBS (Fresenius Kabi USA, Lake Zurich, IL) intraperitoneally (IP) at a dose of 2.3 mg/kg daily, for five days followed by a five-day rest period and another five days of cisplatin injection. Freshly isolated mitochondria were delivered to the mice nasally at 48 and 96 h after the last dose of cisplatin or PBS. To enhance the permeability of the nasal mucosa, 50 U of hyaluronidase (Sigma-Aldrich, St. Louis, MO, USA) in 3 µL was administered per nostril (total of 100 U per mouse) 30 min prior to the nasal administration of mitochondria. For each mouse, up to 170 µg of mitochondria isolated from 4.5E6 human MSC in a total volume of 12 µL were administered at 3 µL per nostril in alternation (total volume 12 µL/mouse).

### Cognitive Assessment

To assess cognitive function, we employed the PBT, NOPRT and Y-maze commencing fourteen days after the last dose of mitochondria.

The PBT for executive functioning consists of three levels of complexity and eleven trials in total as described before 21. In short, mice are placed in a bright compartment (55 cm x 28 cm) connected to a dark compartment (15 cm x 28 cm) by an underpass (4 cm x 2.5 cm). The time taken to enter the dark compartment was recorded in “easy” (open tunnel), “intermediate” (bedding-covered tunnel) and “difficult” (tunnel covered with same color lid) levels. The PBT was conducted over four days - Trials 1 to 3 of the “easy” level on the first day, Trial 4 of the “easy” level and Trials 5 and 6 of the “intermediate” level on the second day, Trial 7 of the “intermediate” level and the “difficult” Trials 8 and 9 on the third day followed by the “difficult” trials 10 and 11 on the fourth day.

The NOPRT to measure the spatial and working memory of mice uses the innate preference of mice for novelty. NOPRT was performed as we previously described 19,21. During training (pre-test), mice were exposed to two identical objects in an arena for 5 min and then returned to their cages for 30 min. In the following 5 min testing phase, mice were exposed to one now-familiarized object in the same location and a novel object placed in a new location. EthoVision XT 10.1 tracking software (Noldus Information Technology Inc., Leesburg, VA, USA) was used quantify time spent with each object and the discrimination index was calculated as per the following equation - (T_Novel_ - T_Familiar_) / (T_Novel_ + T_Familiar_).

The Y-maze test for spatial memory was performed in an arena constituting of three grey plastic, symmetrical 35 cm (length) X 5 cm (width) X 15.5 cm (height) arms, angled at 120° with external spatial room cues, as we previously described 21. Mice were randomly placed in one of the arms and their movement was recorded for 5 min. Exploration of all three arms in sequence before re-entering a previously visited arm was calculated to determine the percentage of perfect alternations.

### Detection of Nasally Delivered Mitochondria in Meninges and Brain

Mice were perfused with ice-cold PBS with 5 U/mL sodium heparin followed by 4% paraformaldehyde and sacrificed at 30 mins, 3 h and 18 h after nasal mitochondria delivery. Brains and skull caps were collected and fixed in 4% paraformaldehyde for 48 h. The meninges were then dissected, incubated in block-perm solution (2% normal goat serum, 1% bovine serum albumin, 0.1% triton-X100 and 0.05% Tween in PBS) for 1 h at room temperature and further stained as free-floating tissue with appropriate antibodies in antibody dilution solution (1% bovine serum albumin, 0.5% triton X-100 in PBS). In case of the brains, 25 µm thick coronal or sagittal sections were taken from each time point. To observe the RMS, sagittal sequential slices of 25 µM were taken at 3 mm from either side of the bregma. Brain sections were incubated in blocking solution (2% bovine serum albumin, 10% normal goat serum and 0.1% saponin in PBS) for 1 h at room temperature and stained with antibodies in antibody staining buffer (2% bovine serum albumin, 2% normal goat serum and 0.1% saponin in PBS) for 2 h at room temperature. The free-floating meninges or brain sagittal sections were incubated with primary antibodies overnight at 4 ˚C: rat anti-CD45 (1:50, #553081, BD Biosciences, San Jose, CA, USA), rabbit anti-F4/80 (1:125, #70076, Cell Signaling, Danvers, MA, USA), rat anti-Lyve-1 (1:50, #14-0443-82, ThermoFisher Scientific, Waltham, MA, USA), mouse anti-human mitochondria (1:400, #ab92824, Abcam, Cambridge, MA, USA), mouse IgG1 Isotype Control (1:400, #ab91353, Abcam, Cambridge, MA, USA), mouse anti-human mtTFA (1:500, #ab119684, Abcam, Cambridge, MA, USA), rabbit anti-GFAP (1:500, #AP32987SU-N, OriGene, Rockville, MD, USA), and the secondary antibodies for 2 h at room temperature: Alexa Fluor 488 Goat-Anti-Rat (1:500, #A11006, ThermoFisher Scientific, Waltham, MA, USA), Alexa Fluor 594 goat anti-rabbit (1:500, #A-11037, ThermoFisher Scientific, Waltham, MA, USA), Alexa Fluor 488 goat anti-rabbit (1:500, #A-11034, ThermoFisher Scientific, Waltham, MA, USA), F(ab')2-goat anti-mouse Qdot® 655 (1:50, #Q-11021MP, ThermoFisher Scientific, Waltham, MA, USA). Sections were incubated with DAPI for 5 min and mounted with FluorSave^TM^ (MilliporeSigma, Burlington, MA, USA) and cover-slipped. The meningeal whole mounts and brain sections were imaged with 20x and 40x objectives using Leica DMI4000 SPE Confocal Microscope (Leica Microsystems GmbH, Wetzlar, Germany) and with 20x, 40x, and 100x objectives using Nikon A1R Confocal Microscope (Nikon Instruments Inc., Melville, NY, USA).

### Evaluation of White Matter Integrity and Synaptic Loss

Mice were perfused with ice-cold PBS and brains were fixed in 4% paraformaldehye for 48 h. 25 µm sections were sliced using the Leica SM2010 R sliding microtome (Leica Biosystems Inc., Buffalo Grove, IL, USA). For Black Gold II staining, cortical sections were mounted onto slides and dried overnight at room temperature. Tissue sections were rehydrated with distilled water for 2 min and immersed in 0.3% Black Gold II (#AG105, MilliporeSigma, Burlington, MA, USA) heated to 60°C for 12 min. Slides were washed with distilled water thrice and then transferred to a 1% sodium thiosulfate solution for 3 min at 60°C. Following additional washes, the sections were dehydrated through a descending series of ethanol solutions and then immersed in xylene. Slides were then cover-slipped with DPX Mountant (Sigma-Aldrich, St. Louis, MO, USA). Bright field images were taken with 4x and 20x objectives using EVOS^®^ FL microscope (ThermoFisher Scientific, AMG, Mill Creek, WA, USA). Percent area and coherency were quantified using ImageJ with the OrientationJ plugin as described before 20.

For synaptophysin staining, free-floating mid-brain sections were washed in PBS then incubated in blocking solution (2% bovine serum albumin, 10% normal goat serum and 0.1% saponin in PBS) for 1 h at room temperature and stained with rabbit anti-synaptophysin (1:500, #AB9272, MilliporeSigma, Burlington, MA, USA) overnight at 4°C followed by Alexa 647 goat anti-rabbit (1:500, # A-21245, ThermoFisher Scientific, Massachusetts, USA) in antibody staining buffer (2% bovine serum albumin, 2% normal goat serum and 0.1% saponin in PBS) for 2 h at room temperature. Sections were incubated with DAPI (1:5000) for 5 min, mounted with FluorSave^TM^ (MilliporeSigma, Burlington, MA, USA) and cover-slipped. Three regions of interest (ROIs) in each of the hippocampal CA1 and CA3 regions were imaged using 40x objective with Nikon A1R Confocal Microscope. The number of punctate structures and sum intensity of the ROI were quantified using Nikon NIS-Elements Advanced Research (Nikon Instruments Inc., Melville, NY, USA).

### TEM of Synaptosomal Mitochondria

Synaptosomes were isolated from whole brain as described previously 21. Briefly, mice were perfused with PBS and brains were homogenized using a glass Dounce homogenizer in a 10% w/v of 0.32 M sucrose in a buffer consisting of 145 mmol/L NaCl, 5 mmol/L KCl, 2 mmol/L CaCl2, 1 mmol/L MgCl2, 5 mmol/L glucose, 5 mmol/L HEPES, pH 7.4. The homogenates were centrifuged at 1000 x g for 10 min at 4°C and the supernatant was diluted 1:1 with 1.3 M sucrose HEPES buffer and centrifuged at 20,000 x g for 30 min at 4°C. The pellet containing isolated synaptosomes was resuspended in electron microscopy grade 2% paraformaldehyde - 2% glutaraldehyde in PBS. 4 mice per treatment group were evaluated and 10-12 images per mouse were taken using the JEOL JEM-1010 transmission electron microscope (JEOL USA Inc., Peabody, MA, USA) and scored blindly. Swollen mitochondria, mitochondria with ruffled mitochondrial membrane or disorganized cristae were identified as atypical mitochondria. Synaptosomes showing distinct membrane ruffling or at least two of the following features - ruffled membrane, disrupted membrane, vesicle leakage, or blebs on the membrane were identified as damaged (Figure S6).

### RNA-sequencing and data analysis

72 h after second dose of mitochondria, mice were perfused with ice-cold PBS and hippocampi were rapidly dissected. Total RNA was extracted using RNeasy MinElute Cleanup Kit (Qiagen, Hilden, Germany). Quality control analysis (Figure S8A) was performed by TapeStation (Agilent Technologies, 2017). 72 bp, paired-end, stranded cDNA library was prepared from extracted RNA using the Stranded mRNA-Seq kit (Kapa Biosystems, Wilmington, MA, USA) and sequenced on Illumina HiSeq 4000. All samples generated > 37.5 million paired end reads (Figure S8B). The first 12 bp of the sequenced reads were trimmed out based on the Phred sequencing quality score and nucleotide content bias analysed using the FASTQC toolkit 81. Trimmed reads were mapped using the STAR toolkit 82 to the mouse reference transcriptome Gencode version M16, and its corresponding reference genome, as well as the human chrM chromosome. The following STAR command was used:

@PG ID:STAR PN:STAR VN:STAR_2.6.1c CL:STAR --runMode alignReads --runThreadN 18 --genomeDir <genome_path> --genomeLoad NoSharedMemory --readFilesIn <left_fastq_path> <right_fastq_path> --outFileNamePrefix <file-prefix> --outMultimapperOrder Random --outSAMtype BAM SortedByCoordinate --outSAMstrandField intronMotif --outSAMattributes All --outSAMprimaryFlag AllBestScore --outBAMcompression -1 --outBAMsortingThreadN 14 --outSAMattrIHstart 0 --outFilterType BySJout --outFilterMultimapNmax 10 --outFilterScoreMinOverLread 0.3 --outFilterMatchNminOverLread 0.3 --outFilterMismatchNoverReadLmax 0.06 --outFilterIntronMotifs RemoveNoncanonical --alignIntronMax 1000000 --alignMatesGapMax 1000000 --alignSJoverhangMin 5 --alignSJDBoverhangMin 3 --alignEndsType EndToEnd --alignSoftClipAtReferenceEnds No --sjdbGTFfile <gtf_path> --quantMode GeneCounts --twopassMode Basic

Uniquely mapped reads to the mouse genome and the human chrM chromosome accounted for more than two-thirds of all sequenced reads in all samples (Figure S8B). Mouse gene relative abundances were estimated for each sample using the Stringtie toolkit 83. After filtering regions of the genome with similarity to mouse ribosomal RNA sequences, all known (and some putative) coding gene relative abundances were re-normalized to sum to a million per sample to generate TPM (Supplementary File 1, Sheet 1) relative abundances. A standard approach for identifying stably expressed genes was chosen 84. Kernel-smoothed density estimation of the TPM values in each sample yielded very similar bimodal empirical density functions for each sample, with two peaks corresponding to stably expressed and lowly expressed / undetectable genes (Figure S9). A conservative threshold of > = 0.5 TPM was used to identify genes that were stably expressed. For each treatment condition, the median expression of each gene was calculated (since the median is more resistant to outlier values than the mean) and the gene was categorized for that treatment condition as stably expressed or lowly expressed / undetected, depending on whether the median value was > = or < 0.5 TPM.

We performed two comparisons: PBS-treated hippocampus versus cisplatin-treated hippocampus; and cisplatin-treated hippocampus samples contrasted with and without mitochondrial treatment. The primary goal of the analysis was to test if transcriptional changes due to cisplatin treatment is partly or fully reversed by mitochondrial treatment. For each comparison, only genes that were stably expressed in at least one of the two conditions being compared were selected for analysis (Supplementary File 1, Sheet 1). The number of such genes is shown in Figure 9 and Table S1.

To assess the effect of cisplatin or of administration of mitochondria to cisplatin-treated mice, we used the SSMD 85 to identify differences for every gene with stable expression in at least one condition for each comparison (Supplementary File 1, Sheet 1). The SSMDs_i_ for each gene was calculated by calculating the difference in means for each condition and dividing by the square root of the sum of variances (assuming covariance to be zero). A small smoothing factor of 0.1 is added to the denominator.


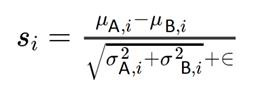


A threshold of > = 0.75 or < = -0.75 was chosen for the SSMD to identify stably expressed genes that were systematically changing between conditions (Supplementary File 1, Sheet 1). Fold changes were calculated (with a smoothing factor of 0.5 added to both the numerator and denominator 86 for every gene with stable expression in at least one condition for each comparison (Supplementary File 1, Sheet 1).

### Ingenuity Pathway Analysis and Functional Analysis

We performed pathway and ontology enrichment analysis using the Ingenuity Pathway Analysis tool (https://www.qiagenbioinformatics.com/products/ingenuitypathway-analysis, Qiagen Inc.) by weighing stably expressed genes with the uncorrected p-value, and filtering out genes which were not stably expressed or which had an uncorrected p-value > 0.1 for each comparison. Pathways with an overlap of < = 10 genes between the pathway gene set and perturbed gene set were discarded, as were pathways with z-score in the range (-2, +2). Disease and function annotations and upstream analysis were also performed using Ingenuity Pathway Analysis (Tables S2 and S3). The relevant functions and upstream regulators were identified by filtering functions or molecules with z-score in the range (-2, +2).

### Statistical Analyses

Data were analysed using GraphPad Prism version 8.0.0 for Windows (GraphPad Software, San Diego, CA, USA) and Matlab (MathWorks^®^, Natick, MA, USA). Two-way ANOVA was performed to test statistical significance followed by two-tailed Tukey's test for post hoc pair-wise, multiple-comparisons.

## Supplementary Material

Supplementary figures and tables.

Supplementary file.

## Abbreviations

MSC: mesenchymal stem cells
PBS: phosphate buffered saline
PBT: puzzle box test
NOPRT: novel object place recognition test
RMS: rostral migratory stream
GFAP: glial fibrillary acidic protein
mtTFA: mitochondrial transcription factor A
TEM: transmission electron microscopy
TPM: transcripts per million
SSMD: strictly standardized mean difference
mTORC2: mammalian target of rapamycin complex 2
NSC: neural stem cells
DAMPs: damage-associated molecular proteins
CNS: central nervous system
CSF: cerebrospinal fluid
Nfe2l1: nuclear factor, erythroid 2 like 1
IP: intraperitoneally
ROIs: regions of interest
