# Supplementary material for: Nasal administration of mitochondria reverses chemotherapy-induced cognitive deficits
Source: Theranostics. 2021 Jan 1;11(7):3109–30. doi: 10.7150/thno.53474 (PMC7847685; doi:10.7150/thno.53474)
Supplement: Supplementary file 1 — Supplementary figures and tables. [file thnov11p3109s1.pdf]

Nasal Administration of Mitochondria Reverses Chemotherapy-Induced Cognitive Deficits

Jenolyn F. Alexander, Alexandre V. Seua, Luis D. Arroyo, Pradipta Ray, Andi Wangzhou, Laura Heiß-Lückemann,

Manfred Schedlowski, Theodore Price, Annemieke Kavelaars, Cobi J. Heijnen

SUPPLEMENTARY FIGURES AND TABLES

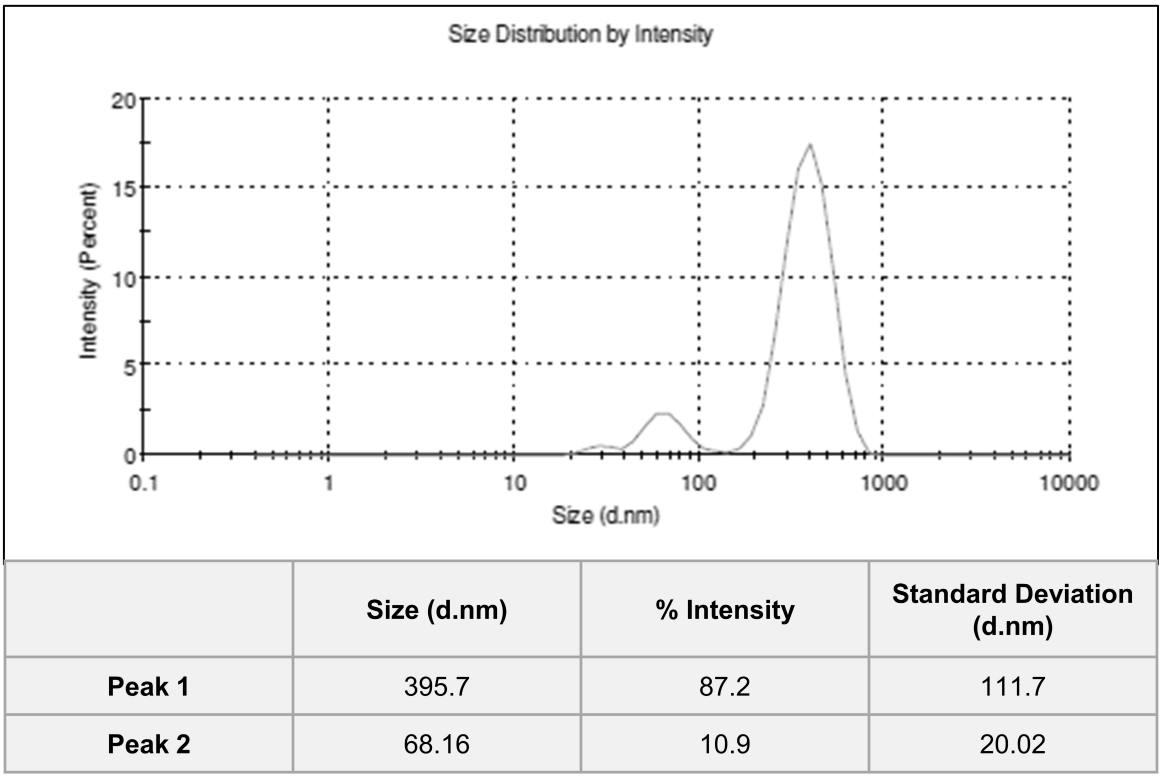

	Size (d.nm)	% Intensity	Standard Deviation (d.nm)
**Peak 1**	395.7	87.2	111.7
**Peak 2**	68.16	10.9	20.02

Figure S1. Average size of mitochondria isolated from human MSC

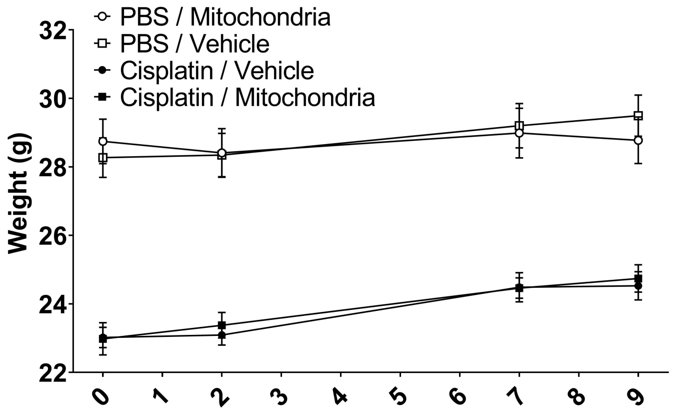

Figure S2. Body weight measurements of all treatment groups after nasal administration of mitochondria

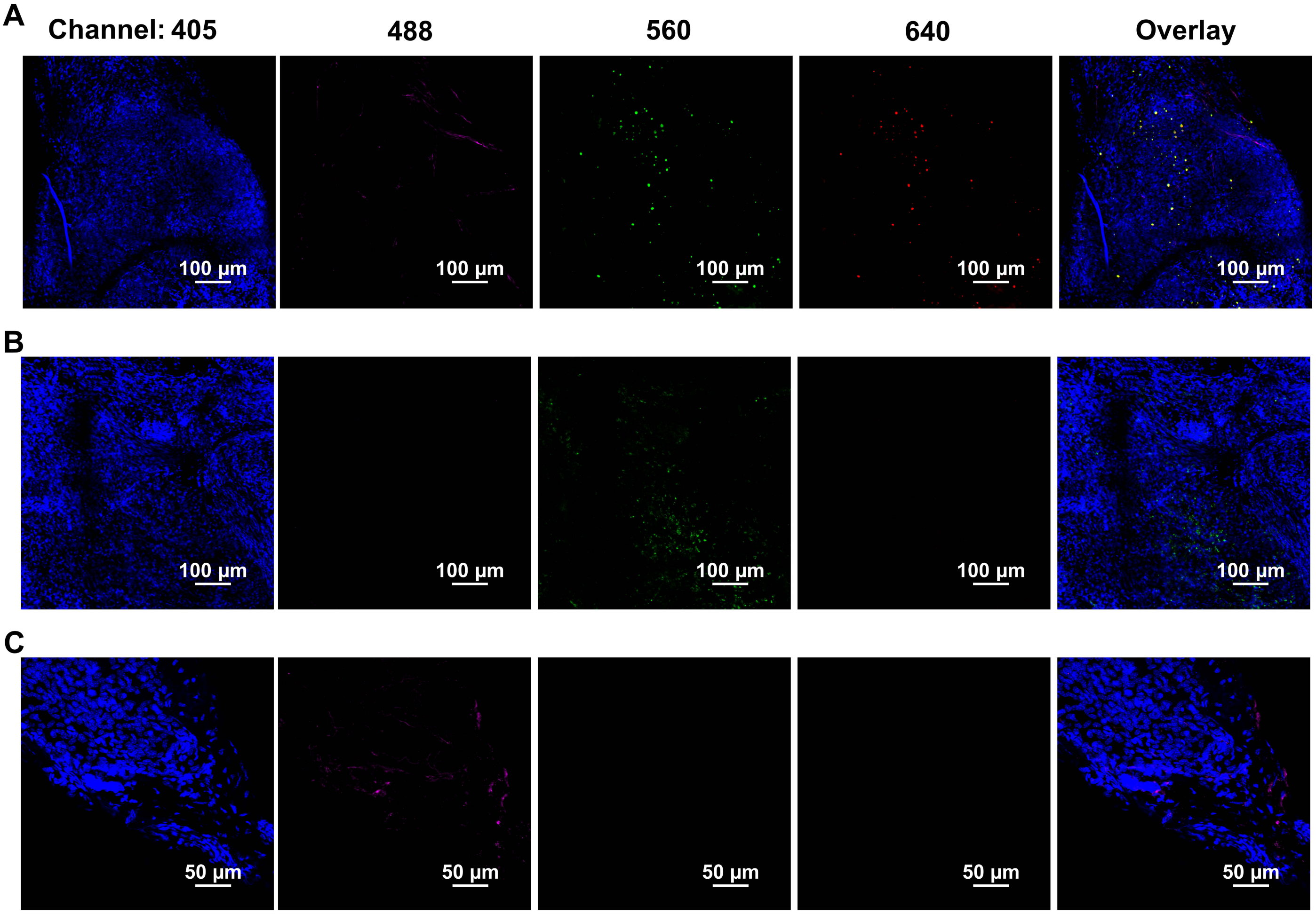

Figure S3. No autofluorescence and no labeling of mitochondria with control IgG in meninges. (A) Individual channels of meningeal olfactory region showing DAPI $^{+}$ nuclei (405 channel), Lyve-1^{+} lymphatic vessels but no mitochondria-specific signal (488 channel), DsRed^{+} mitochondria (560 channel), anti-human mitochondria^{+} mitochondria (640 channel) and merged channels. (B) IgG isotype control for the anti-human mitochondria antibody showing DAPI^{+} nuclei (405), no autofluorescence (488), DsRed^{+} mitochondria (560), IgG control (640) and merged channels. The results show that there is no co-localization of the DsRed signal with the 488 signal or the signal from the control IgG antibody; scale bar 100 µm. (C) Negative control showing DAPI^{+} nuclei (405 channel), Lyve-1^{+} lymphatic vessels (488 channel), no DsRed $^{+}$ signal (560), no anti-human mitochondria^{+} signal (640) and merged channels in cisplatin-treated mouse that did not receive mitochondria; scale bar 50 µm. Images taken with 20x objective.

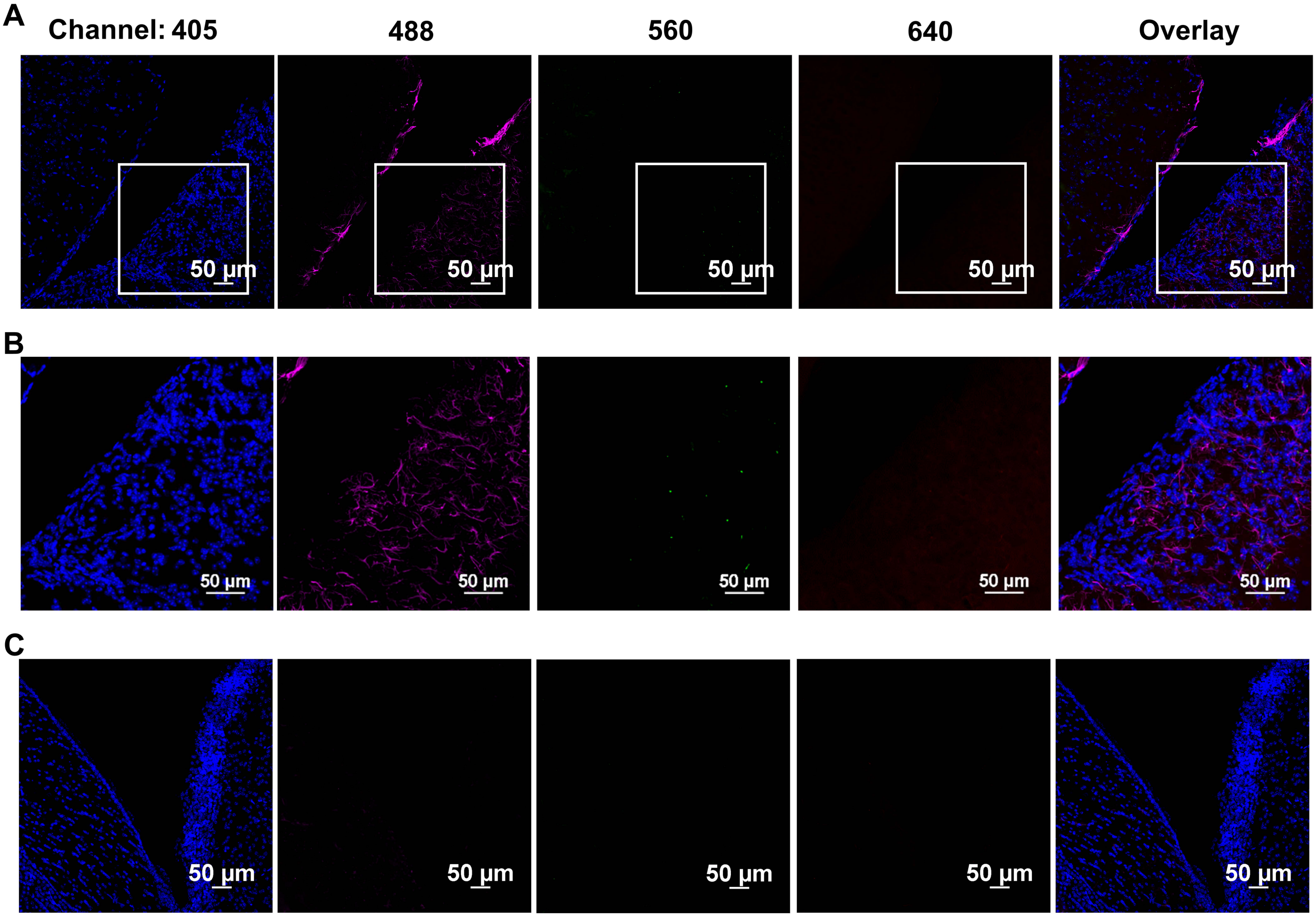

Figure S4. No autofluorescence and no labeling of mitochondria with control IgG in brain. (A) IgG isotype control for the anti-human mitochondria antibody in brain and (B) their corresponding enlarged images showing DAPI$^+$ nuclei (405), GFAP$^+$ cells (488), DsRed$^+$ mitochondria (560), IgG control (640) and merged channels. The results show that there is no co-localization of the DsRed signal with signal from the control IgG antibody. (C) Negative control showing DAPI$^+$ nuclei (405 channel), no autofluorescence (488 channel), no DsRed$^+$ signal (560), no anti-human mitochondria$^+$ signal (640) and merged channels in cisplatin-treated mouse that did not receive mitochondria; scale bar 50 µm. Images taken with 20x objective.

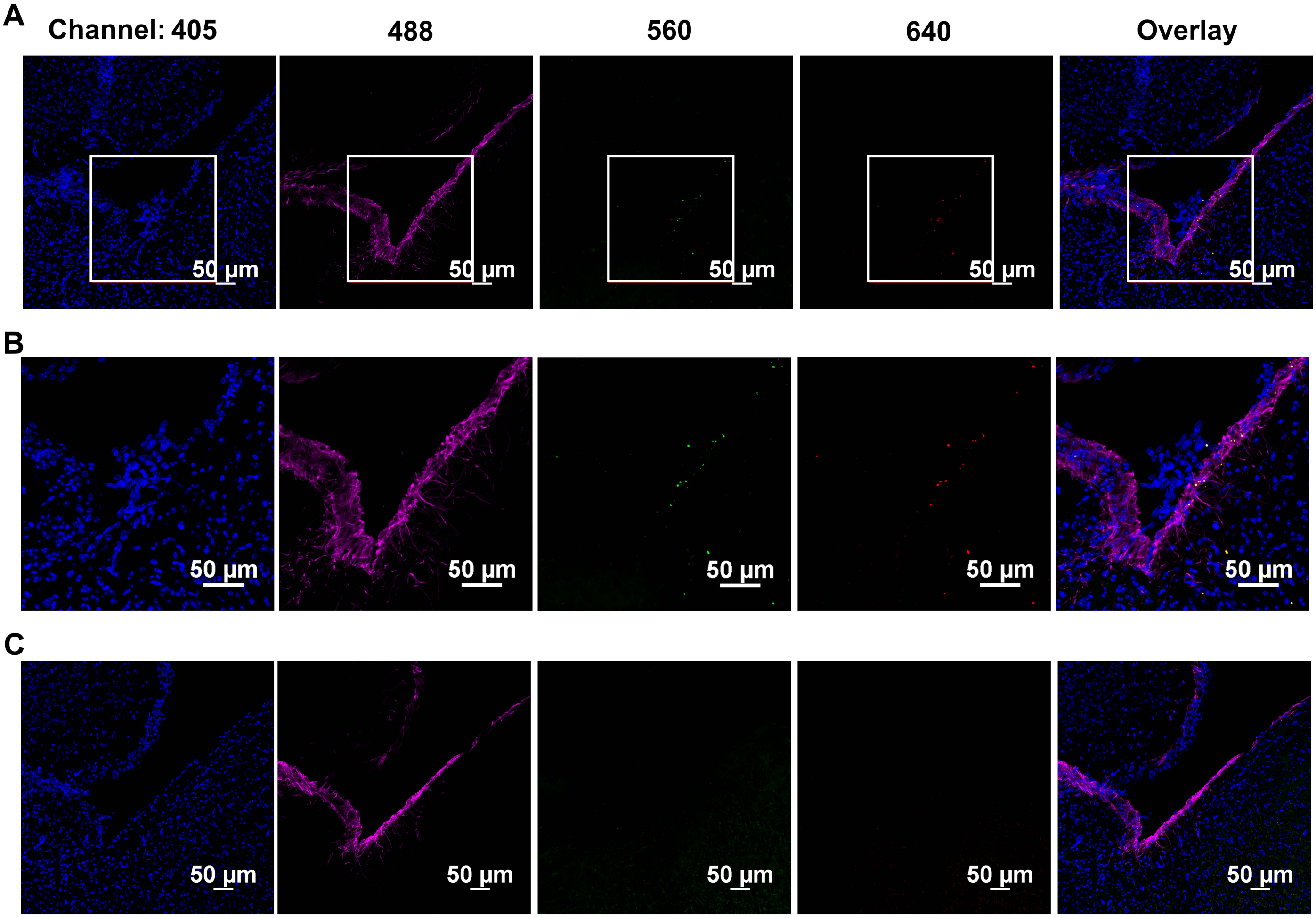

Figure S5. Colocalization of DsRed⁺ mitochondria with anti-human mitochondrial TFAM antibody. (A) Individual channels of brain with (B) corresponding magnified images showing DAPI $^{+}$ nuclei (405 channel), GFAP^{+} glial limitans (488 channel), DsRed^{+} mitochondria (560 channel), anti-human mtTFA^{+} mitochondria (640 channel) and merged channels which depict colocalization of DsRed and anti-human mtTFA^{+} signals. (C) Negative control - brain of vehicle control mouse showing DAPI $^{+}$ nuclei (405), GFAP^{+} glial limitans (488), no DsRed^{+} mitochondria (560) no anti-human mtTFA^{+} mitochondria (640) and merged channels; scale bar 50 µm. Images taken with 20x objective.

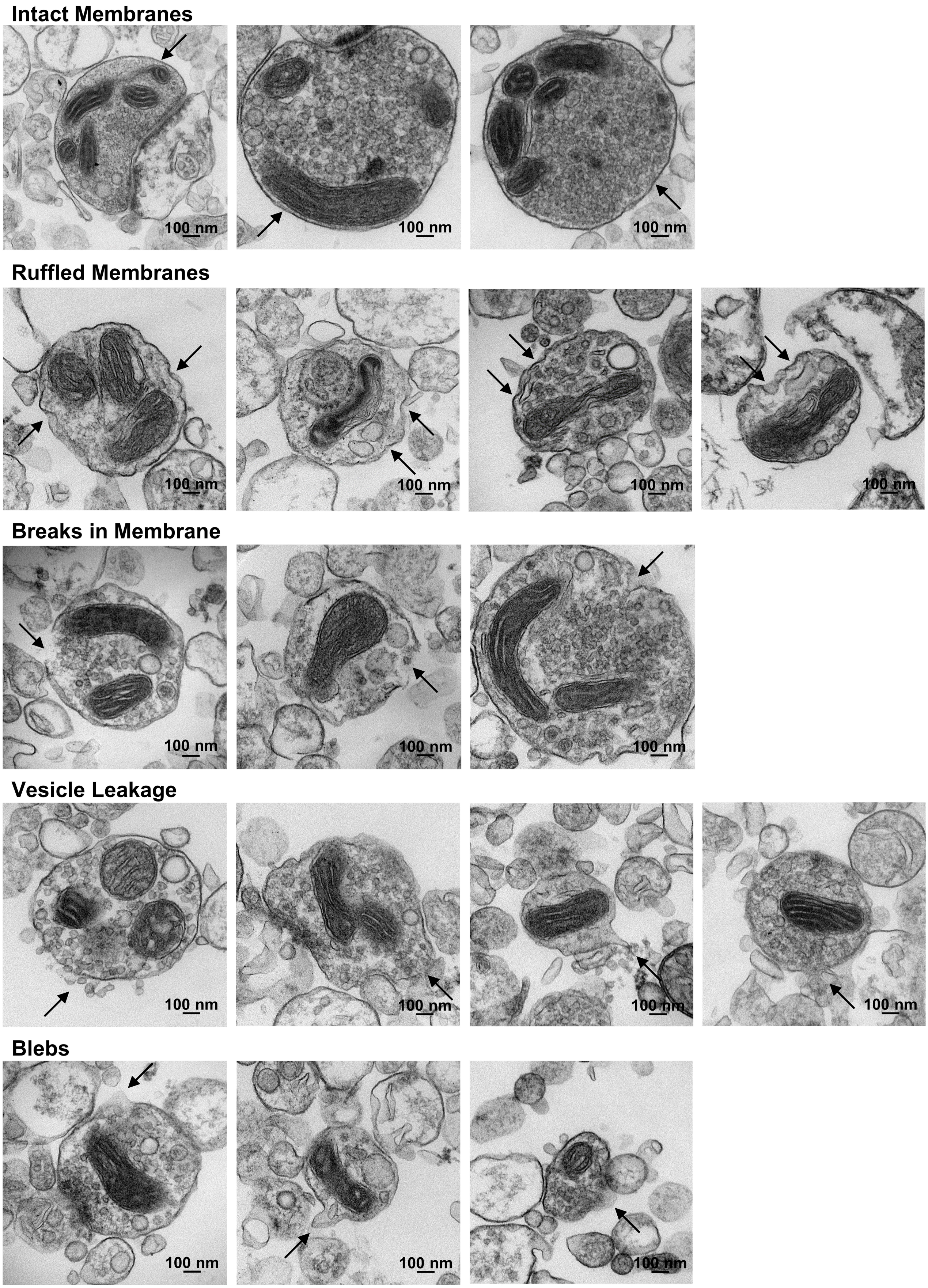

Figure S6. Criteria for identifying damaged synaptosome membrane

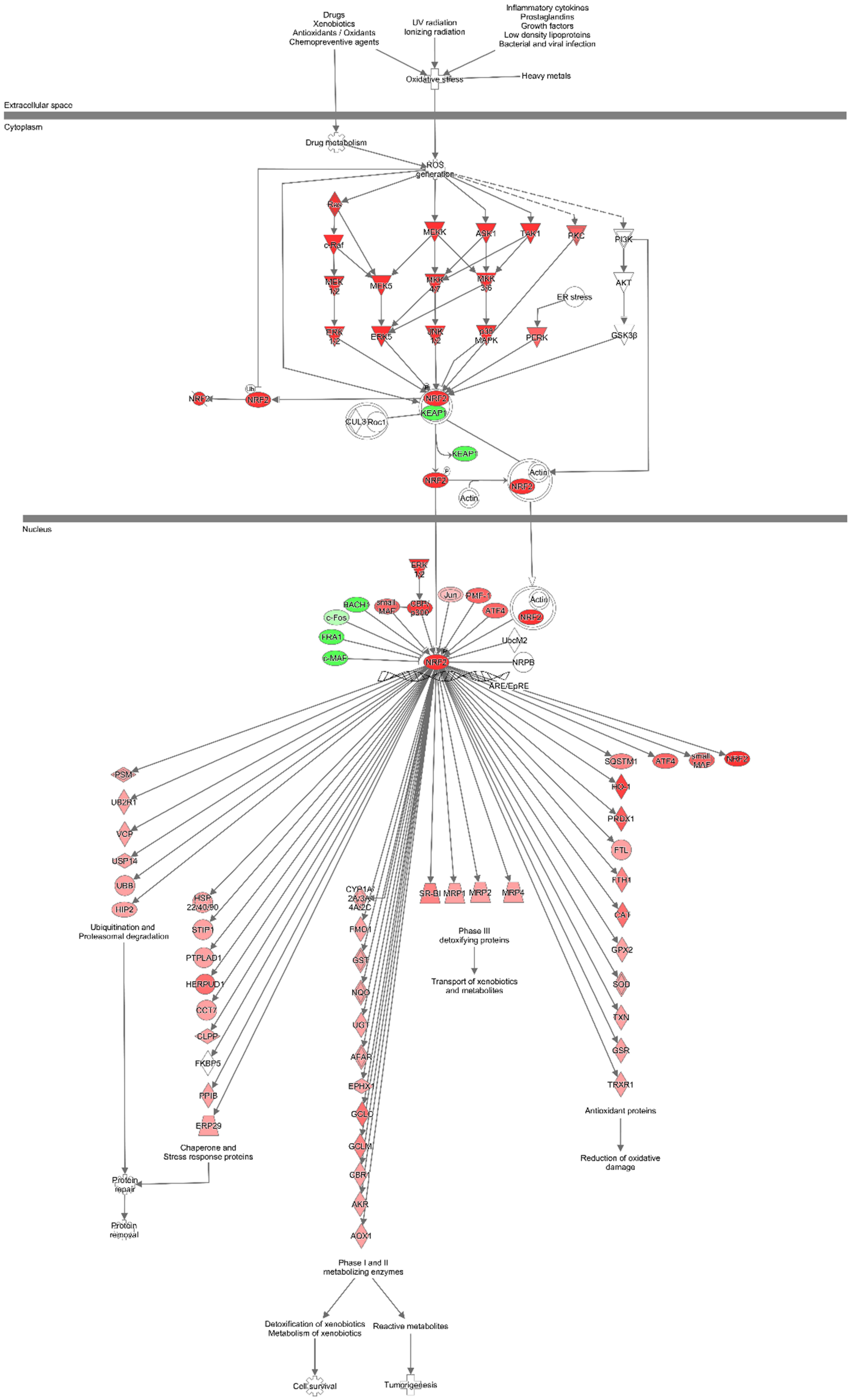

Figure S7. Expected activation state of *Nrf2*-mediated oxidative stress response as shown by IPA

A

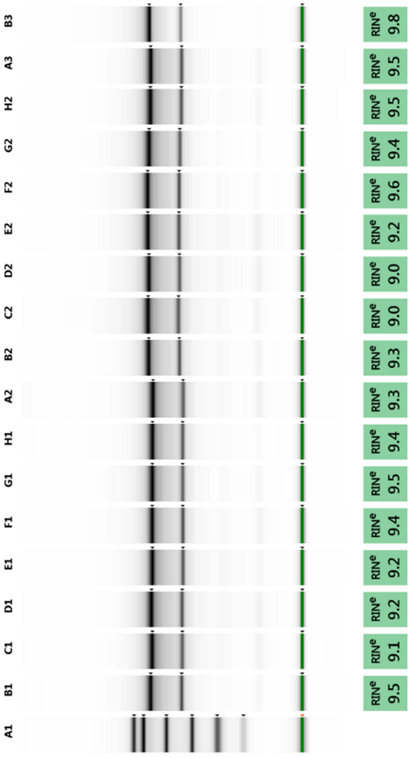

Sample description	Repli cate #	SMF Volume ul	SMF Qubit Conc ng/ul	SMF Total Quantity ng	RIN/RQN	DV200
Control						
Hippocampus+Cisplatin+Mito	4	14	1278.0	17892.0	9.5	97.3
Hippocampus+Cisplatin+Mito	3	14	1422.0	19908.0	9.5	97.5
Hippocampus+Cisplatin+Mito	2	14	1662.0	23268.0	9.4	97.6
Hippocampus+Cisplatin+Mito	1	14	1824.0	25536.0	9.6	97.6
Hippocampus+PBS+Mito	4	14	1284.0	17976.0	9.2	94.4
Hippocampus+PBS+Mito	3	14	1314.0	18396.0	9	95.6
Hippocampus+PBS+Mito	2	14	912.0	12768.0	9	96.1
Hippocampus+PBS+Mito	1	14	1170.0	16380.0	9.3	97.2
Hippocampus+Cisplatin	4	14	1182.0	16548.0	9.3	94.8
Hippocampus+Cisplatin	3	14	1044.0	14616.0	9.4	98
Hippocampus+Cisplatin	2	14	1170.0	16380.0	9.5	97.6
Hippocampus+Cisplatin	1	14	1140.0	15960.0	9.4	96.3
Hippocampus+PBS	4	14	978.0	13692.0	9.2	96.7
Hippocampus+PBS	3	14	978.0	13692.0	9.2	96
Hippocampus+PBS	2	14	1194.0	16716.0	9.1	96.5
Hippocampus+PBS	1	14	1110.0	15540.0	9.5	98.2
Ladder						

Default image (contrast 50%). Image is scaled to sample. (Generated Nov 29, 2018)

B

Treatment	Replicate	# sequenced reads	% uniquely mapped reads
Hippocampus+ PBS	1	43,351,335	86.83
Hippocampus+ PBS	2	38,772,804	85.49
Hippocampus+ PBS	3	42,099,495	86.39
Hippocampus+ PBS	4	39,234,197	78.1
Hippocampus+ Cisplatin	1	38,679,875	82.99
Hippocampus+ Cisplatin	2	44,986,438	85.97
Hippocampus+ Cisplatin	3	38,193,418	80.73
Hippocampus+ Cisplatin	4	41,714,148	83.43
Hippocampus+ PBS+Mito	1	48,909,561	67.57
Hippocampus+ PBS+Mito	2	47,195,869	86.46
Hippocampus+ PBS+Mito	3	44,284,048	85.16
Hippocampus+ PBS+Mito	4	42,510,611	82.23
Hippocampus+ Cisplatin +Mito	1	38,406,982	87.1
Hippocampus+ Cisplatin +Mito	2	44,538,606	88.01
Hippocampus+ Cisplatin +Mito	3	37,871,612	85.46
Hippocampus+ Cisplatin +Mito	4	38,548,657	86.14

Figure S8. Quality control analysis of extracted RNA for RNA-sequencing

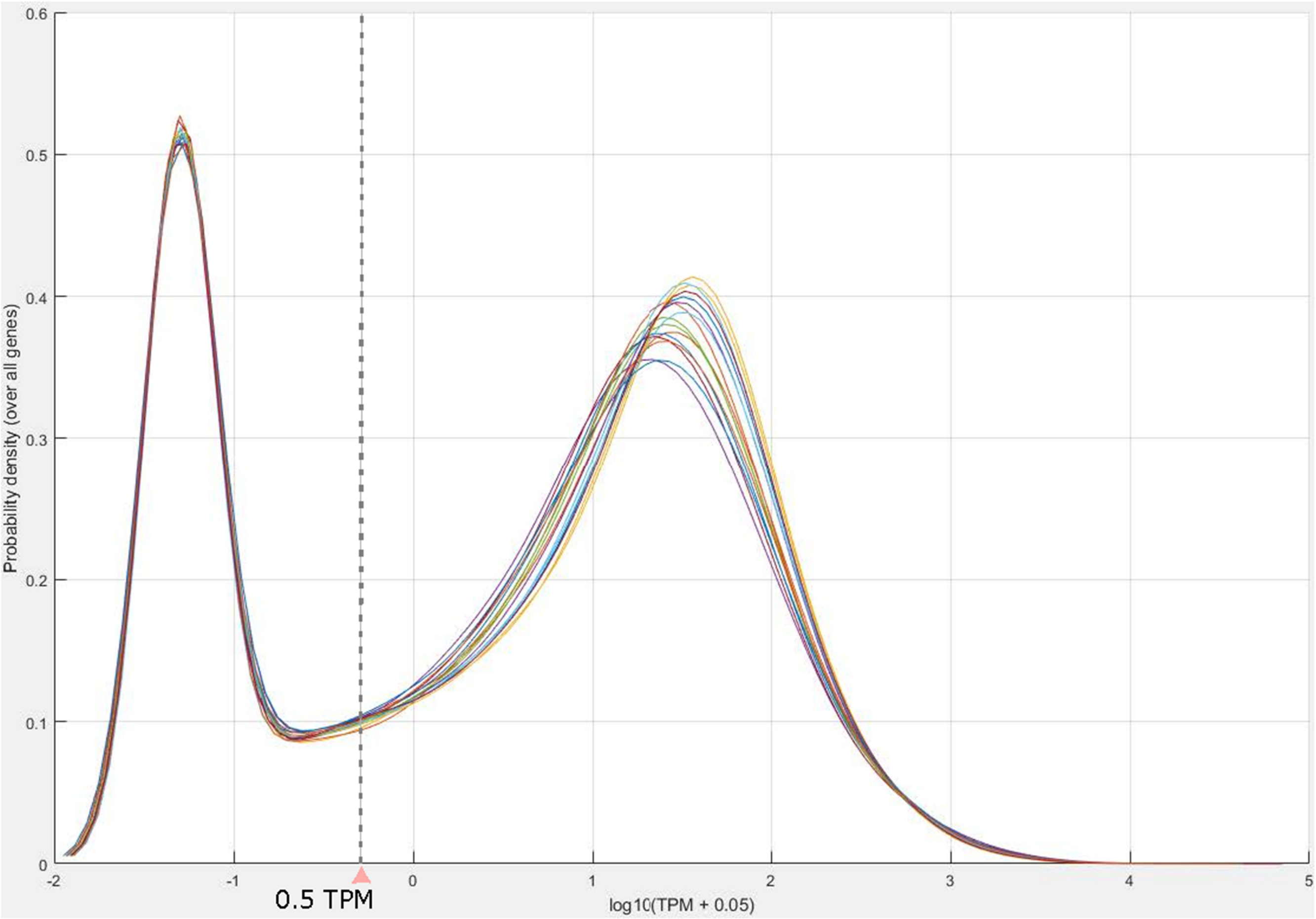

Figure S9. Identification of stably expressed genes

	S1	S2	S3	S4	C1	C2	C3	C4	SM1	SM2	SM3	SM4	CM1	CM2	CM3	CM4
**Gene Abundance (TPM > 0.0)**	16130	15887	16074	15760	15891	16527	15843	15929	16109	16164	16188	16000	15919	16129	15841	15978
**Stably Expressed Genes (TPM >= 0.5)**	14741	14566	14792	14394	14582	14956	14569	14600	14853	14836	14848	14676	14619	14821	14489	14611

	Cisplatin vs PBS	Cisplatin + Mitochondria vs Cisplatin
**Stably Expressed Genes (TPM >= 0.5)**	14784	14736
**Systematically Changed Genes (SSMD >= 0.75)**	2673	2396
**Systematically Differentially Expressed Genes (Fold Change <1/1.2 and > 1.2)**	1813	1308

Table S1. Gene abundances, stably expressed and systematically differentially expressed gene values quantified

Categories	Function	Diseases or Functions Annotation	Predicted Activation State	p-value	Activation z-score
Nervous System Development and Function	Coordination	Coordination	Increased	2.50E-05	2.427
Cell Morphology, Cellular Assembly and Organization, Function and Maintenance	Formation	Formation of cellular protrusions	Increased	5.40E-07	2.304
DNA Replication, Recombination and Repair	Metabolism	Metabolism of DNA	Increased	4.66E-05	2.223
Cellular Development, Growth and Proliferation, Nervous System Development and Function, Tissue Development	Neurogenesis	Neurogenesis of neuroepithelial cells	Increased	4.67E-05	2
Cellular Assembly and Organization	Organization	Organization of organelle	Decreased	1.23E-05	-2.138
Developmental Disorder	Hypoplasia	Hypoplasia	Decreased	3.05E-05	-2.754
Neurological Disease	Movement Disorder	Movement Disorders	Decreased	3.56E-06	-3.319
Psychological Disorders	Hyperactive Behavior	Hyperactive behavior	Decreased	3.99E-05	-3.413
Neurological Disease	Motor Dysfunction or Movement Disorder	Motor dysfunction or movement disorder	Decreased	1.22E-06	-3.869
Organismal Survival	Morbidity or Mortality	Morbidity or mortality	Decreased	1.91E-18	-4.585
Organismal Survival	Organismal Death	Organismal death	Decreased	5.50E-18	-4.755

Table S2. Relevant functions predicted to be regulated by nasal administration of mitochondria in cisplatin-treated mice, as revealed by Ingenuity Pathway Analysis

Upstream Regulator	Molecule Type	Predicted Activation State	Activation z score	P-Value of Overlap
Tcf7l2	transcription regulator	Activated	3.433	0.00943
Alpha Catenin	group	Activated	3.207	0.00138
Rictor	other	Activated	2.93	0.000782
Dnmt3b	enzyme	Activated	2.828	0.414
Ngf	growth factor	Activated	2.816	2.12E-06
Cd40	transmembrane receptor	Activated	2.705	0.0849
Ep400	other	Activated	2.63	0.00926
Hsf1	transcription regulator	Activated	2.49	2.14E-05
Cr1l	other	Activated	2.449	0.0088
Il4	cytokine	Activated	2.341	0.00119
H1-6	other	Activated	2.333	0.0576
H1f1	other	Activated	2.333	0.0576
Jak1/2	group	Activated	2.309	0.00066
Ybx1	transcription regulator	Activated	2.273	0.00245
Aldh1a2	enzyme	Activated	2.236	0.0341
let-7	microRNA	Inhibited	-2.22	0.00583
Pkc(s)	group	Inhibited	-2.231	0.127
Efna5	kinase	Inhibited	-2.236	0.0423
Efna4	kinase	Inhibited	-2.236	0.0907
Efna1	other	Inhibited	-2.236	0.316
Efna3	kinase	Inhibited	-2.236	0.098
Hic1	transcription regulator	Inhibited	-2.236	0.271
Sparc	other	Inhibited	-2.267	0.0481
Hbb-b1	transporter	Inhibited	-2.309	0.00679
Cd44	other	Inhibited	-2.345	0.0158
Tgfbr1	kinase	Inhibited	-2.557	0.00032
Brd4	kinase	Inhibited	-2.63	0.00425
mir-181	microRNA	Inhibited	-2.828	0.00425
mir-1	microRNA	Inhibited	-2.892	0.0223
miR-1-3p (and other miRNAs w/seed GGAAUGU)	mature microRNA	Inhibited	-2.957	0.0277

Table S3. Top 15 upstream regulators predicted to be activated and top 15 predicted to be inhibited by nasal administration of mitochondria to cisplatin-treated mice by Ingenuity Pathway Analysis
